# RL-MLZerD: Multimeric protein docking using reinforcement learning

**DOI:** 10.3389/fmolb.2022.969394

**Published:** 2022-08-26

**Authors:** Tunde Aderinwale, Charles Christoffer, Daisuke Kihara

**Affiliations:** ^1^ Department of Computer Science, Purdue University, West Lafayette, IN, United States; ^2^ Department of Biological Sciences, Purdue University, West Lafayette, IN, United States

**Keywords:** protein docking, multiple protein docking, reinforcement learning, docking order prediction, protein bioinformatics

## Abstract

Numerous biological processes in a cell are carried out by protein complexes. To understand the molecular mechanisms of such processes, it is crucial to know the quaternary structures of the complexes. Although the structures of protein complexes have been determined by biophysical experiments at a rapid pace, there are still many important complex structures that are yet to be determined. To supplement experimental structure determination of complexes, many computational protein docking methods have been developed; however, most of these docking methods are designed only for docking with two chains. Here, we introduce a novel method, RL-MLZerD, which builds multiple protein complexes using reinforcement learning (RL). In RL-MLZerD a multi-chain assembly process is considered as a series of episodes of selecting and integrating pre-computed pairwise docking models in a RL framework. RL is effective in correctly selecting plausible pairwise models that fit well with other subunits in a complex. When tested on a benchmark dataset of protein complexes with three to five chains, RL-MLZerD showed better modeling performance than other existing multiple docking methods under different evaluation criteria, except against AlphaFold-Multimer in unbound docking. Also, it emerged that the docking order of multi-chain complexes can be naturally predicted by examining preferred paths of episodes in the RL computation.

## Introduction

Proteins interact with other proteins when they perform biological functions in a cell. Therefore, knowing how proteins physically interact with each other is an essential step toward molecular- and atomic-level understanding of functional mechanisms of proteins. A three-dimensional (3D) picture of individual proteins and protein complexes can be determined by biophysical experiments such as X-ray crystallography and cryogenic electron microscopy. However, experimental methods are often time-consuming and expensive. Moreover, determining the structures of multimeric protein complexes is often found to be extremely difficult. As a result, there are still many important protein complex structures in the human proteome and the proteomes of other organisms which are not yet determined by experiment.

To supplement experimental approaches, computational protein docking methods have been studied extensively in the past few decades (Dominguez et al., 2003; Aytuna et al., 2005; Venkatraman et al., 2009; Kundrotas and Vakser, 2010; Moal and Bates, 2010; Kozakov et al., 2017). The progress of protein docking prediction has been regularly monitored by the community-wide assessment, the Critical Assessment of PRedictions of Interactions (CAPRI) (Lensink et al., 2020). Despite the steady progress of the field as observed in CAPRI, the majority of developments have focused on pairwise docking. Methods for assembling three or more chains have been receiving less attention.

CombDock (Inbar et al., 2005) was the first method which performed multimeric protein docking. In CombDock, first pairwise docking of individual subunits is computed. Then, hierarchical combinatorial assembly of subunits is performed to build a full complex structure. Our group developed Multi-LZerD (Esquivel-Rodríguez et al., 2012), which constructs multimeric protein complex models using a genetic algorithm from pairwise solutions modelled by LZerD (Venkatraman et al., 2009). These two methods can model hetero complexes where each chain is different. There are also multiple protein docking methods that are limited to symmetric assembly (Popov et al., 2014) (Ritchie and Grudinin, 2016) (Schneidman-Duhovny et al., 2005) (Pierce et al., 2005). These methods use user input of the symmetry type to identify binding interface in an input structure and assembles multiple copies of the input structure into a complex.

The major challenge of docking multiple chains is the combinatorially larger number of possible docking conformations compared to pairwise docking cases. In multimeric protein docking, different patterns of interacting subunit pairs (topologies) need to be considered, since not all subunit pairs are in contact in a complex. Symmetric assembly is more tractable than asymmetric assembly because it has additional constraint in generating complexes but still has more modeling steps than pairwise docking.

Recently, the appearance of AlphaFold (Jumper et al., 2021), which made a notable breakthrough in single-chain protein structure prediction (Pereira et al., 2021) has enabled new methods for multimeric protein assembly. ColabFold (Mirdita et al., 2021) adapted AlphaFold for multi-chain docking by connecting the sequence of each chain with linker regions and folding the whole complex as if it were a single protein structure. AlphaFold-Multimer (Evans et al., 2021) uses most of the original deep learning framework of AlphaFold, which was retrained specifically for multiple chain protein docking. In their manuscript posted on bioRxiv (Evans et al., 2021), it was applied to complexes with two to three chains.

In this work, we developed a new multiple-chain docking method, Reinforcement Learning for Multimeric protein docking with LZerD (RL-MLZerD). RL-MLZerD approaches the problem in a similar manner as our previous work, Multi-LZerD, where precomputed pairwise docking poses (decoys) are selected and assembled into complex structures. The novelty of this work is the adaptation of RL to perform the search of the conformation space. We found that RL is better suited for exploring the docking space than the genetic algorithm used in Multi-LZerD because RL is able to identify individual correct decoys through multiple episode runs, which can effectively shrink the search space as the number of runs is increased. Also, since an assembly episode can resemble the physical process of protein complex formation, the assembly order of multiple chain complexes can be directly predicted based on assemble path that is favored during docking. RL-MLZerD was tested on a dataset of 30 protein complexes with three to five chains. The average root mean square deviation (RMSD) of the best assembled model across all targets is 2.50 Å and 6.30 Å for bound and unbound docking, respectively. RL-MLZerD showed a better performance when compared against CombDock and Multi-LZerD for both bound and unbound cases. We further compared RL-MLZerD against AlphaFold-Multimer and ColabFold. In bound cases, RL-MLZerD outperformed ColabFold, we observed similar performance between the AlphaFold-Multimer and RL-MLZerD. However, for unbound cases AlphaFold-Multimer showed better modeling accuracy against RL-MLZerD. Finally, we show that the assembly order of complexes can be predicted with 76.5% accuracy by RL-MLZerD when tested on complexes in the dataset that has the assembly order information.

## Materials and methods

### Dataset construction

We benchmarked RL-MLZerD on 30 multimeric protein complexes. These protein complexes were selected from PDB (Berman, 2000) using the following criteria: First, we selected complexes with 3–5 chains in the biological assembly confirmed in the PISA server (Krissinel and Henrick, 2007). Complexes with DNA/RNA and those with a short subunit of fewer than 10 residues were removed. Complexes were grouped if any chain in one had over 25% sequence identity to any chain in another. Subunits of each complex were rotated and shifted randomly from the docked conformations in the PDB file to remove potential bias to the starting conformation. The PDB entry list is provided in Supplementary Table S1.

### Pairwise docking

RL-MLZerD builds protein complex models in two steps. First, it constructs a pool of pairwise decoys for each pair of chains. In the second step, the pool of pairwise decoys is searched and different combinations of decoys are explored as assembly episodes in the RL framework. The resulting assembled models are evaluated and rewards are propagated in the Q-table of RL, which records preferred decoys with high probability scores, to guide efficient searches of assembly.

In the pairwise docking stage, LZerD was used to generate candidate decoys for each pair of chains. LZerD generates hundreds of thousands of models for an input structure pair, which were clustered to reduce similar solutions. Two decoys were clustered if they had a Cα RMSD of 4.0 Å or smaller. We used this cluster criterion because it worked well in past rounds of CAPRI (Christoffer et al., 2020). A representative decoy with the best LZerD docking shape score was selected for each cluster (Venkatraman et al., 2009). Then, decoys were ranked by the sum of score ranks (the ranksum score) (Peterson et al., 2018a) with GOAP (Zhou and GOAP, 2011), DFIRE (Zhou and Zhou, 2009) and ITScorePro (Huang and Zou, 2011).

For a target, we generated three sets of random poses of individual subunits and generated pairwise decoys using LZerD for each set separately. Each of the three sets of decoys were ranked by the ranksum score, and the top 333, 333, and 334 decoys from each of the three sets, respectively, were combined to construct a pool of 1,000 pairwise decoys. The 1,000 decoys for each subunit pair were then used for the RL multiple docking phase.

### Assembling pairwise decoys with reinforcement learning

**FIGURE 1:**
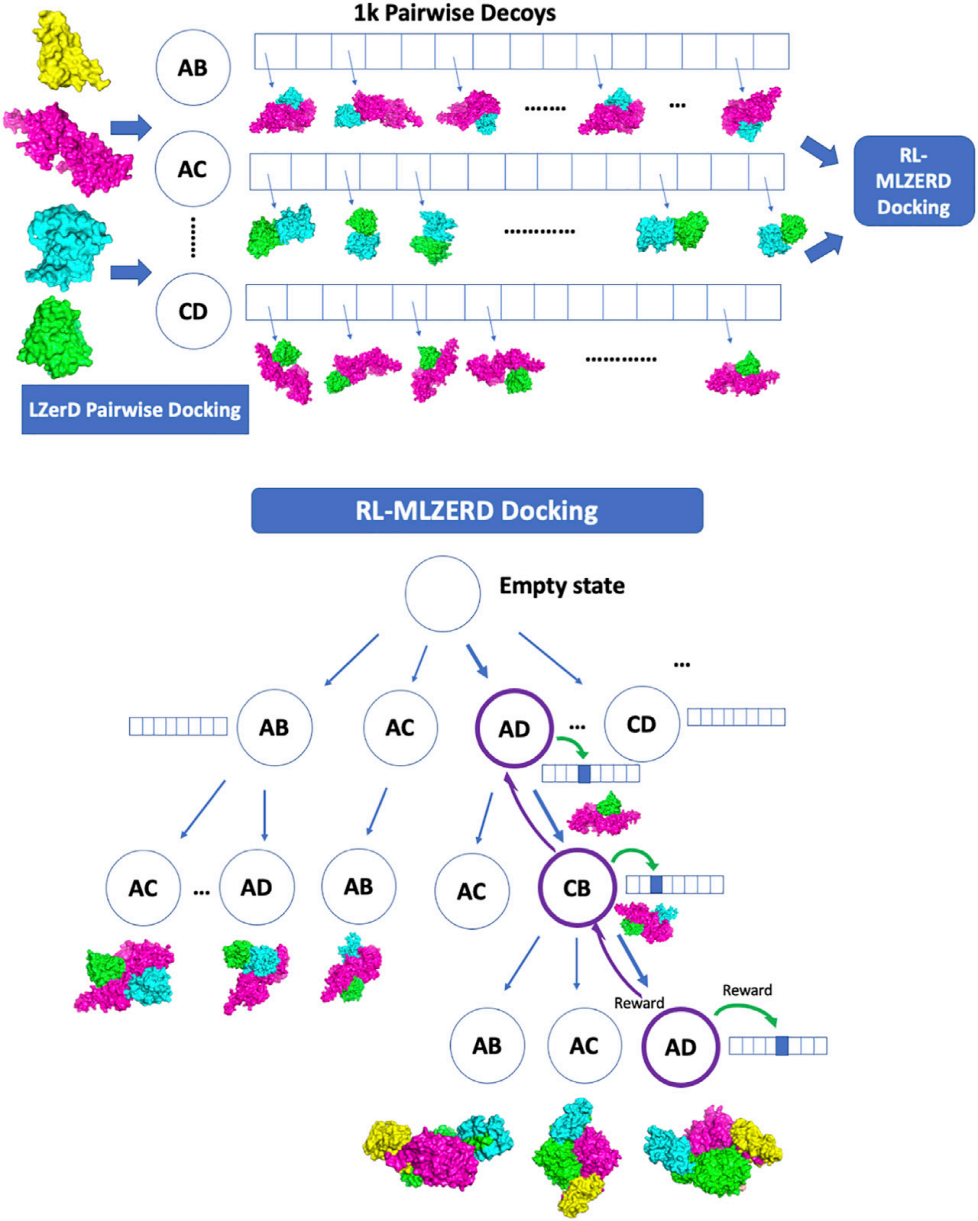
Overview of RL-LZerD. In the first step, pairwise docking of every pair of subunits is performed using LZerD. 1000 decoys are selected for each pair. In the subsequent step, pairwise decoys are assembled using reinforcement learning. When an assembly of full subunit complex is successful without too many clashes, reward maybe propagated back to assembly states and decoy states used along the episode.

**FIGURE 2:**
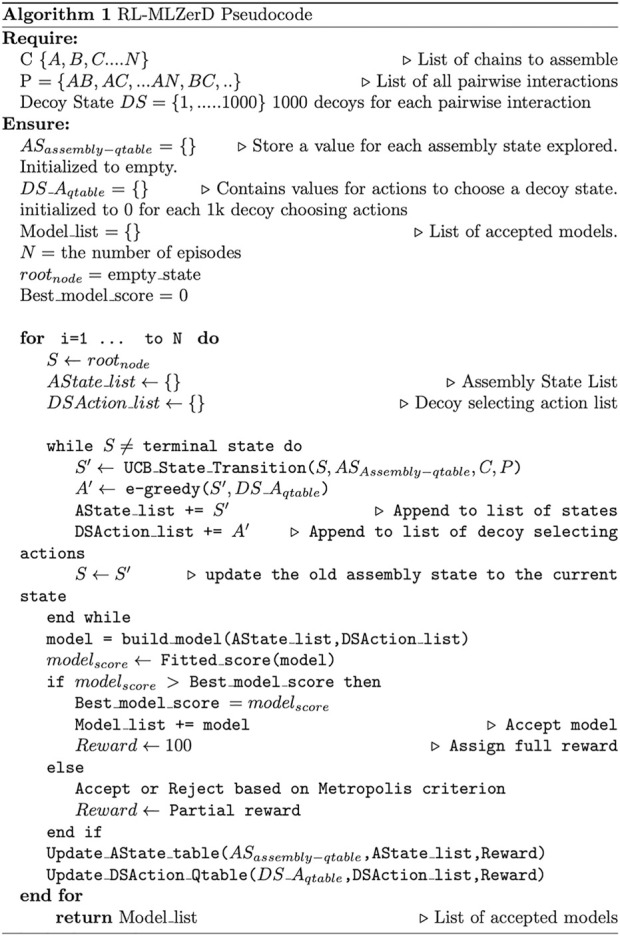
Pseudocode of RL-MLZerD. For each episode, *AState_list* represents the assemble state visited for the episode, *DSAction_list* represents the selected actions (decoys) for each decoy state. The *build_model* function takes the *AState_list* and *DSAction_list* and returns the assembled model. *Fitted_score* computes a score of a model. *Update_state_table* updates the state value estimate for the assemble state that participated in building the model, *update_action_qtable* uses the Bellman Fold equation to update the decoy state actions value estimate that participated in the model building.

Generated pairwise decoys were assembled with the RL framework shown in Figure 1. The pseudo-code is provided in Figure 2. A docking process is represented by an episode in the RL, which makes choices of two types of states along the path. The first one is called assembly states (circles in the diagram in Figure 1), which denote subunit combinations, e.g., AD > AC (adding C to the complex by choosing a decoy of the AC pair). Starting from an empty state at the top of the diagram, subunits are added one at a time by choosing a pairwise decoy until a terminal state is reached, where all subunits are assembled. The second type of states is called decoy states (an array of boxes in Figure 1), which denotes decoys in the pool generated for each subunit pair. Thus, an episode consists of a set of selections of assembly states and a decoy state at each assembly state.

Accordingly, there are two types of actions, one that selects the next assembly state and the other that specifies a decoy from a decoy pool for a subunit pair. The next assembly state is selected with the Upper Confidence Bound (UCB) policy (Kocsis and Szepesvári, 2006), which tries to balance exploitation and exploration with the following score:

$$v_{i}+C\times \sqrt{\frac{\ln N}{n_{i}}}$$ (1)

where v_i_ is the value of assemble state i, C is a hyper-parameter, which was set to 1.0 at the beginning and slowly reduced by 1.0/(the total number of episodes) after each episode is performed. N is the total number of visits to the parent state of the assemble state i, n_i_ is the number of visits to the assembly state i. When N is small at the early stage of computation, states are selected mainly by v_i_, while less-visited states are more explored as N becomes larger. At the beginning of the computation when values are not accumulated yet, the next assembly state is randomly chosen.

The second type of action deals with the decoy state. Each time an assembly state is visited, a decoy state is selected from a pool using ε-greedy (Tokic, 2010) as the policy. The ε-greedy policy dictates that the agent exploits the best action (i.e. the decoy with the highest shape score) among possible choices most of the time (i.e., 1-ε) but randomly select a decoy state with probability ε. We set ε to 1.0 at the beginning and slowly reduced it by 1.0/(the total number of episodes) after each episode was performed.

Here we briefly walk through the algorithm (Figures 1, 2). The docking procedure starts from a root node in Figure 1, and the next assembly state is selected according the UCB policy, followed by a selection of a decoy state. This procedure is iterated until the full complex is built. The developed full chain complex model is evaluated by a scoring function that is a linear combination of four scoring terms, a molecular mechanics potential (Esquivel-Rodríguez et al., 2012), the solvent accessible surface area, the radius of gyration (RG), and atom clash counts. RG is included to encourage compact assemblies. An atom clash is recognized if two heavy atoms are closer than 3 Å to each other. Weights of the terms were determined by a logistic regression classifier with 4-fold cross validation trained on complex models of two quality classes, with an interface RMSD (as defined in the CAPRI evaluation (Lensink et al., 2020)) less than or over 4.0 Å.

During an episode, a partially-assembled complex is checked for atom clashes due to the newly selected action at the decoy state. If the resulting partially-assembled complex at the state has a number of atomic clashes higher than the threshold (n−1) * 100, where n is the number of assembled protein chains, the newly selected decoy state is rejected and replaced with a different decoy that is ranked among the top 5 by Q score until an acceptable model is found. The modeling process moves on to the next assembly state to add the next subunit if the top 5 decoy selections still result in a model with a high number of atomic clashes.

Once an episode generated a complex model, the model evaluation score is checked to see if it has the best score that has been discovered thus far from all episodes. This signifies a newly discovered model with the best score, and a such model is assigned a full reward of 100 points. Any other model score that falls short of this criterion is assigned a partial reward if the Metropolis criterion is met:

$$P=\exp\left(\;\frac{-\Delta E}{{\;k}_{b}T}\right)$$ (2)

Where ΔE is the score difference between the new model and the current best model. We report the results with a normalization factor, $k_{b}T$ , which was set to 6.0. A complex model is accepted if P is larger than a random number generated between 0 and 1. As a variation of the method, we also report results where we set P to a constant value of 0.6, thereby giving a constant 60% chance of acceptance for assembled model regardless of the model score.

If a model score is close to the current best, it has a high chance to be accepted even if it is lower than the best. If the model is accepted, a partial reward is assigned by discounting the 100 points based on the calculated probability to reflect the difference between the current best score and the score of the new model. On the other hand, a reward of 10 points is provided if the model does not pass the metropolis criterion. This is because the model is geometrically possible (if it is not rejected by high number of atom clashes) and thus we do not want to penalize the path that generated the model. A penalty reward of −2 is assigned if the model is rejected due to a high number of atomic clashes. Thus, unlike conventional RL methods where the goal is fixed, the goal of RL-MLZerD is constantly being updated based on the current best discovered model.

Parameters used in RL, namely, the normalization factor (Eq. 2) set to 6.0, the full reward set to 100, the reward of 10 given to a rejected model, and the penalty of −2, were determined by a small number of tests on a couple of targets. To examine how the different parameters affect to the results, in Supplementary Table S2 we examined an exhaustive 108 combinations of these four parameters on nine targets. The parameter values did not affect to the modeling results for two targets. It turned out that the parameters we selected were the most common parameter values individually that worked best across the nine targets.

At last, we explain how values are updated in the RL framework. Values for assembly states, $v_{i}$ used in Eq. 1, in eligible states are updated at the end of an episode as follows:

$$v_{i}={\;v}_{i}+\eta \left[r_{t}\right],\;n_{i}=n_{i}+1$$ (3)

Where η is the learning rate, set to 1.0; $r_{t}$ is the reward assigned to the complex model at the end of the episode (e.g., 100 points). $n_{i}$ is the number of times the assembly state was visited. The eligible states that are updated are those which participated in the model building path of the episode. According to Eq. 3, the same reward value is added to all the eligible states along the episode.

The reward obtained at the end of the episode is also propagated to decoy states selected. The update is based on the temporal difference using the Bellman-Ford Equation (Sutton and Barto, 1998):

$${\text{New}\;\text{DS}}_{\text{Aqtable}}\left(s,a\right)={\;\text{DS}}_{\text{Aqtable}}\left(s,a\right)+\alpha \left[R\left(s,a\right)+\gamma \max{\text{DS}}_{\text{Aqtable}}\left(s^{\prime },a^{\prime }\right)-{\;\text{DS}}_{\text{Aqtable}}\left(s,a\right)\right]$$ (4)

where s is the assembly state, a is the selected action (decoy) for the assembly state. $\text{New}\;\text{DS}\_A_{\text{qtable}}\left(s,a\right)$ is the updated Q score for the assembly-decoy state pair, $\text{DS}\_A_{\text{qtable}}\left(s,a\right)$ is the old Q score, α is the learning rate, where we use an adaptive learning rate which decays from 1.0 to 1.0 * 0.85^(episode/1000)^ after every 1000 episodes. R(s,a) is the reward. For a terminal state, it is the reward given to the episode and it is 0 for all intermediate states. γ is the discount rate for future rewards, which was set to 0.25. $\max\;\text{DS}\_A_{\text{qtable}}\left(s^{\prime },a^{\prime }\right)$ is the maximum score among decoys $\left(a^{\prime }\right)$ of the next state visited s’.

Typically, 1500 to 12,000 models are generated for a target complex. They are clustered by LB3DClust (Terashi et al., 2012), and are then ranked by the sum of score ranks by the scoring function mentioned above and the VoroMQA score (Olechnovič and Venclovas, 2017). For each cluster, the best scoring model was selected as the representative.

### Running Multi-LZerD and CombDock

Multi-LZerD was run using the same pairwise decoy set as RL-MLZerD. As for CombDock, we ran it three times each with one of the same three random poses of subunits. The three runs for any given target produced a combined 100–300 models. The models were gathered and ranked by their docking scores, which were written in output files named combdock.results. Thus, the input subunit structures of Multi-LZerD and CombDock were the same as RL-MLZerD for both bound and unbound docking results.

## Results

We first discuss results of bound cases, where individual subunit structures were extracted from the PDB files. Next, we report docking results of unbound cases where the starting structures were altered from bound cases. Finally, we discuss the implications of reinforcement learning for predicting assembly paths of complexes.

### Bound docking results

The docking results on bound cases are summarized in Table 1. The best (smallest) RMSD among the top 5 scored models was reported for each target. For evaluating the performance for a target, we mainly considered the best model among the top 5 ranked models following the CAPRI evaluation. In parentheses after this RMSD, we also provided the best RMSD achieved among all the models generated before clustering. The total number of models generated is provided in Supplementary Table S3.

**TABLE 1:** RMSD of models for the bound docking cases.

PDB-ID	RLMZD	RLMZD-M	M-LZerD	CombDock
1A0R (3)^a^	0.87 (0.87)^b^	0.87 (0.87)	1.0 (0.87)	21.53 (17.75)
6GWJ (3)	0.88 (0.81)	2.48 (0.81)	0.88 (0.88)	15.51 (10.52)
1VCB (3)	1.38 (1.33)	1.31 (1.31)	1.06 (0.93)	14.67 (11.30)
1A6A (3)	0.74 (0.74)	0.74 (0.74)	0.74 (0.74)	13.04 (13.04)
1IOD (3)	6.33 (1.85)	5.32 (1.85)	5.32 (1.85)	17.44 (10.09)
1NVV (3)	0.94 (0.94)	0.94 (0.94)	1.25 (0.90)	10.82 (10.82)
4YX7 (3)	1.60 (1.60)	1.64 (1.64)	1.65 (1.26)	7.56 (7.56)
2H47 (3)	8.75 (1.97)	5.84 (4.43)	11.16 (2.49)	20.71 (16.13)
2GD4 (3)	1.14 (1.14)	19.16 (5.70)	8.83 (8.66)	8.47 (8.47)
2ASS (3)	3.92 (3.92)	3.92 (3.92)	4.73 (1.26)	22.55 (18.02)
1P3Q (3)	1.37 (1.37)	10.66 (3.12)	11.05 (5.4)	8.2 (8.2)
1JSU (3)	0.84 (0.84)	18.93 (3.51)	0.88 (0.88)	14.81 (14.81)
1EPT (3)	1.23 (1.23)	1.78 (1.78)	5.45 (5.45)	16.52 (14.95)
1RHM (4)	1.67 (1.67)	1.47 (1.47)	1.43 (1.43)	17.58 (17.58)
1NNU (4)	1.47 (1.47)	20.69 (5.26)	2.75 (1.47)	17.95 (17.16)
1QGW (4)	14.19 (9.26)	3.77 (3.77)	19.06 (11.07)	21.84 (20.02)
1CYD (4)	1.66 (1.66)	0.90 (0.90)	0.87 (0.87)	21.14 (16.92)
1IZB (4)	1.03 (0.95)	1.03 (1.02)	9.56 (1.02)	11.79 (9.35)
6MWR (4)	25.27 (2.6)	23.09 (3.02)	25.66 (21.28)	25.06 (23.92)
3LL8 (4)	1.31 (1.21)	1.31 (1.21)	8.9 (1.16)	23.33 (22.22)
4IHH (4)	10.12 (10.1)	32.71 (16.70)	35.85 (12.98)	34.38 (28.53)
6RLX (4)	8.59 (5.45)	8.31 (4.84)	8.65 (7.11)	12.13 (10.64)
1GL2 (4)	6.64 (2.22)	10.28 (4.52)	26.38 (15.19)	16.49 (13.36)
3UAI (4)	10.8 (2.97)	11.63 (2.60)	1.01 (1.01)	33.49 (16.76)
1D1I (5)	7.92 (1.27)	7.75 (4.58)	8.22 (2.18)	15.15 (13.93)
1CT1 (5)	1.32 (1.31)	1.68 (1.60)	1.50 (1.46)	19.60 (14.24)
1CN3 (5)	1.42 (1.15)	1.22 (1.15)	1.36 (1.36)	24.61 (11.62)
1W85 (5)	7.43 (4.76)	7.35 (4.19)	18.64 (7.25)	32.74 (27.92)
4FTG (5)	1.80 (1.80)	1.39 (1.30)	1.18 (1.18)	11.34 (11.34)
4RT4 (5)	6.44 (6.44)	12.25 (8.71)	10.19 (8.70)	15.80 (12.91)
Avg. (Å)	**4.64 (2.50)**	7.35 (3.25)	7.83 (4.27)	18.21 (15.50)
≤8.0 Å^c^	**24 (28)**	20 **(28)**	17 (24)	1 (1)

RLMZD, RL-MLZerD with a fixed P of 0.6; RLMZD-M, RL-MLZerD with Metropolis criterion; M-LZerD, Multi-LZerD. The best (smallest) RMSD of top 5 ranked models are reported.

a The number of chains of the complex is shown in the parentheses with a PDB ID.

b The numbers shown in the parentheses is the best RMSD among all the models generated before clustering by the method. The total number of models generated is provided in Supplementary Table S3.

c The number of targets with RMSD ≤ 8.0 Å is counted.

Best values in a row are shown in bold.

As mentioned in the previous section, we designed two variations of RL-MLZerD, one that used a fixed acceptable probability (P) of 0.6 in Eq. 2 (RLMZD in Table 1) and the other that used the Metropolis criterion with a $k_{b}T$ value of 6.0 (RLMZD-M). On average, the former (with the fixed P) had a lower RMSD value of 4.64 Å than the latter, which had 7.35 Å. When RMSDs of individual targets were compared, the two versions of RL-RMSD showed a lower RMSD than the other for the same number of cases, 12 cases each. We also counted the number of targets which yielded a model with an RMSD less than 8.0 Å, to distinguish the models where the complexes have essentially same interaction modes between chains. RL-MLZerD had 24 (80.0%) such cases while RL-MLZerD-Metropolis had 20 (67%) cases. Therefore, overall, RL-MLZerD showed better performance compared to RL-MLZerD-Metropolis. The modeling accuracy did not have apparent correlation with the secondary structure content of target complexes (Supplementary Figure S1). Furthermore, the presence of an ion at the interface of the protein complex did not affect the modeling accuracy of RL-MLZerD with statistical significance (Supplementary Table S4).

To understand how effective RL was in identifying the best combinations of pairwise decoys, in Supplementary Table S5 we compared the best (lowest) RMSD generated by RL-MLZerD with the best possible RMSD achievable from the pairwise decoy pool used. The best possible RMSD for a complex target was approximated by selecting the best RMSD model from exhaustive combinations of five best pairwise decoys of each pair. For 83.3% (25/30) cases, a model within 1.0 Å RMSD was generated by RL-MLZerD with an average RMSD difference of 0.79 Å.

Next, we compared the two versions of RL-MLZerD with Multi-LZerD and CombDock, two existing methods that are designed for modeling hetero multimer complexes. Models by Multi-LZerD were assembled from the same pairwise decoy sets as RL-MLZerD, which were generated from three random pose subunit poses. In terms of the average RMSD among the top 5 models, RL-MLZerD (with *p* = 0.6) had the lowest value, 4.64 Å, followed by RL-MLZerD-Metropolis (7.35 Å), and then Multi-LZerD (7.83 Å), and CombDock was the last (18.21 Å). This order is consistent when the best RMSD value among all generated models (shown in the parentheses in Table 1) was considered.

**FIGURE 3:**
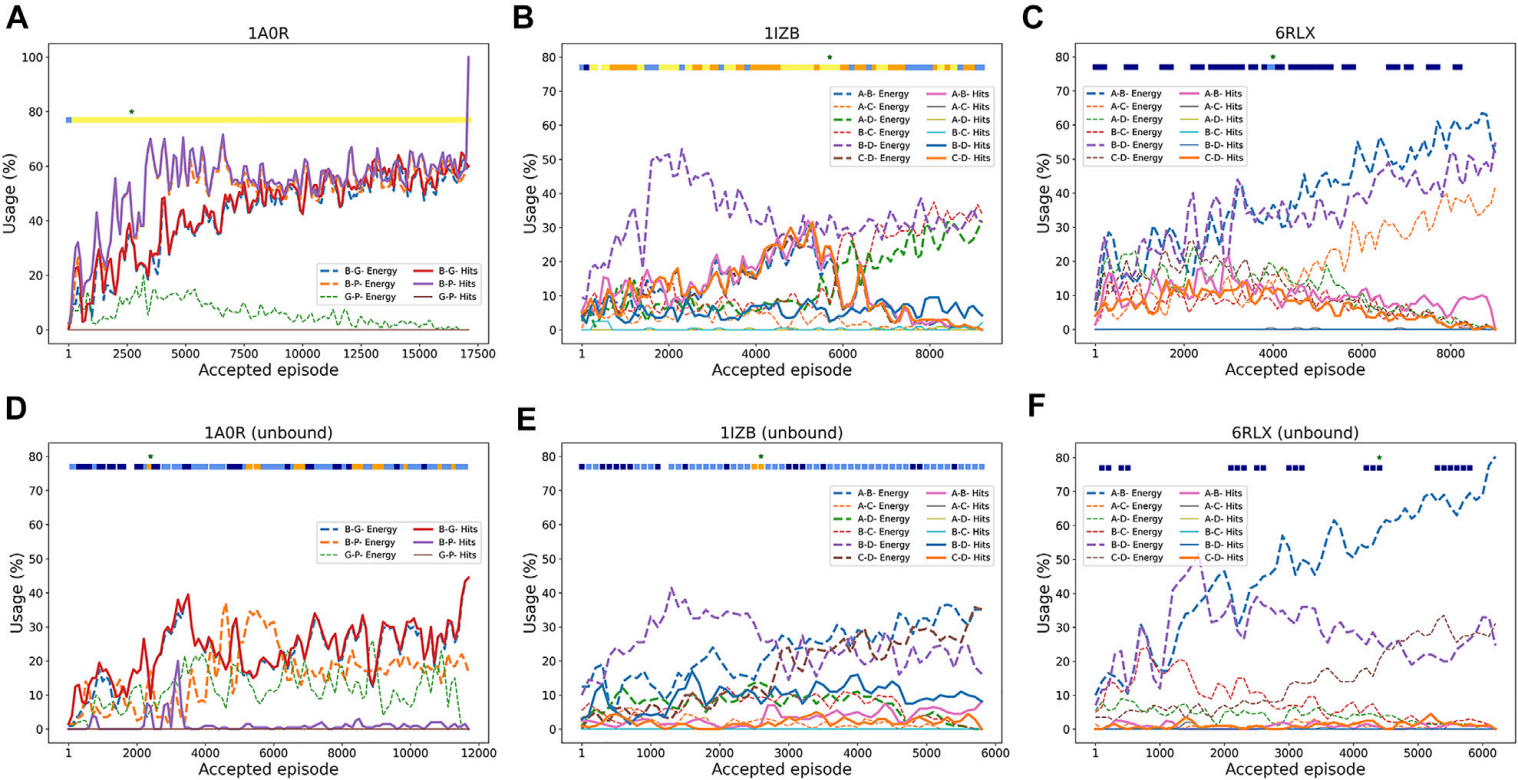
Examples pairwise decoy usage in episodes in RL. In the three target examples, we showed the fraction of pairwise decoys with an RMSD less than 4.0 Å (called hits, e.g., A-B Hits for hits for A-B chain complex) that were selected within a window of 200 accepted episodes. We also counted decoys that were within the top 10 energy ranking (called Energy, e.g., A-B Energy), which have a low energy among other decoys (but not necessarily with a low RMSD). The percentage of the use of a decoy was averaged by a window of 100 episodes. Episodes that were terminated due to many clashes were not counted. The green star “*” at the top of the panel represents the episode where the best RMSD model was found. The colored square boxes at the top of each plot represents RMSD ranges of models generated at along the episodes: yellow, 0–2 Å; orange 2–4 Å; light blue, 4–6 Å; dark blue, 6–8 Å; empty, > 8 Å **(A)**, 1A0R (a 3 chain complex; bound case); **(B)**, 1IZB (a 4 chain complex; bound case); **(C)**, 6RLX (a 4 chain complex; bound case); **(D)**, 1A0R (unbound); **(E)**, 1IZB (unbound); **(F)**, 6RLX (unbound).

To understand how RL worked, in Figure 3 we analyzed how RL selected pairwise decoys along episodes. We examined how often good decoys (called hits), i.e. pairwise docking models within a 4.0 Å RMSD from the native, and the top 10 best energy models were selected from decoy pools of each subunit pair. The first example (Figure 3A) is a successful three-chain complex target, 1A0R, where we obtained a near-native model of 0.87 Å. For this target, hits for all three chain pairs were increasingly more selected as the episode progressed, indicating the RL algorithm successfully prioritized the hits. The color bar at the top of the plot is colored almost entirely in yellow except for the start of the episodes, indicating near-native models with a less than 2.0 Å RMSD were generated throughout the process. The next example (Figure 3B) showed an interesting history of episodes. In this four-chain complex, hits for A-B (pink) and C-D (orange) were increasingly selected to reach a usage of 30% until around the 5500th episode, which guided successful model generation of low RMSD values (color bar). The best RMSD model was constructed at this point as shown with the star. However, after that point, decoys of different chain pairs that have a low energy (dashed lines) but structurally incorrect (not hits) have dominated the usage, which led to models with a worse RMSD. In the last example (Figure 3C), the RL increased the usage of low energy decoys as designed as more episodes were run; however, the modeling was not successful because low energy decoys were not hits. Thus, this is a problem of the energy used for evaluating decoys.

**FIGURE 4:**
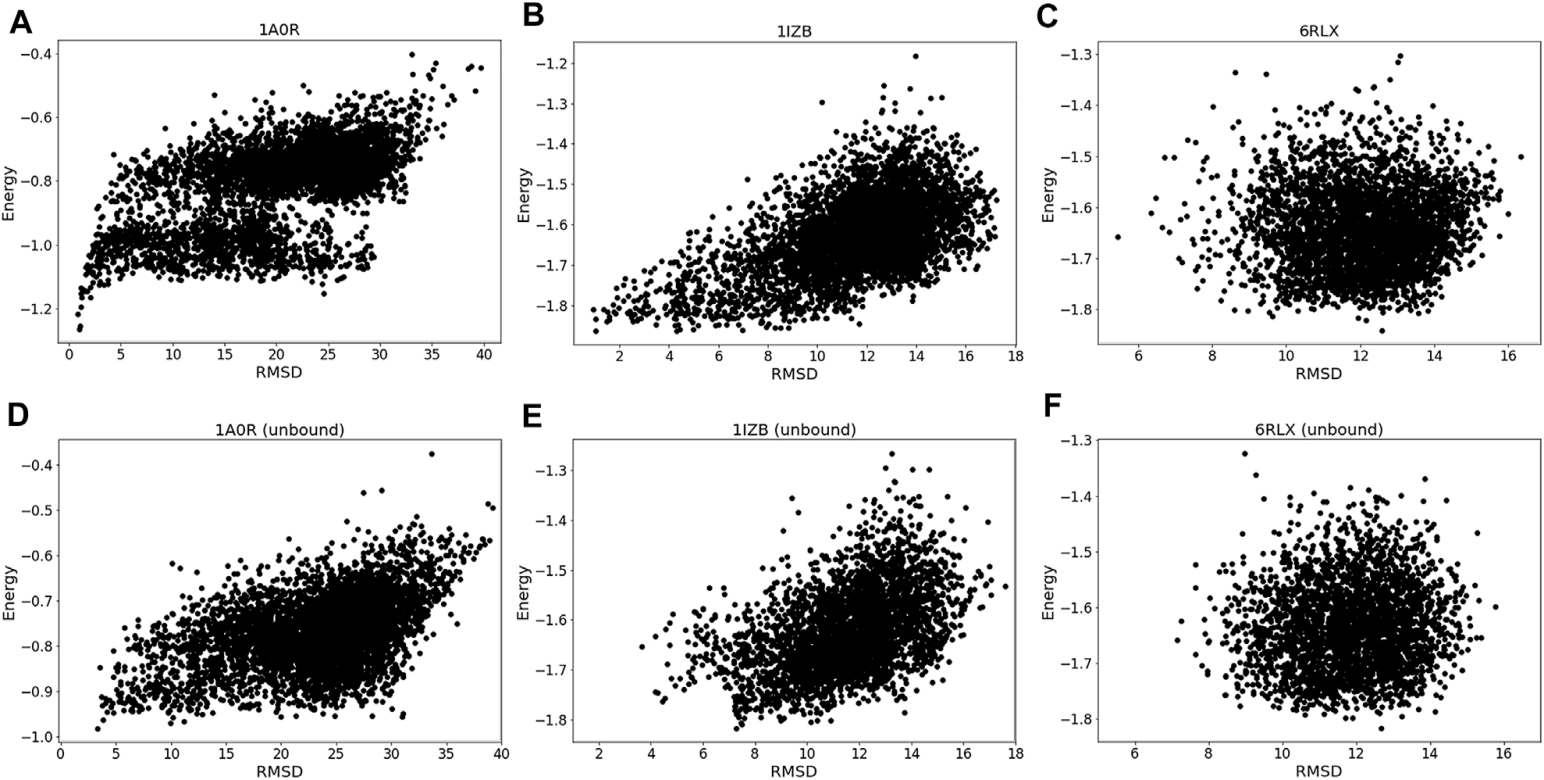
Energy distribution vs. RMSD. The x-axis shows the model RMSD while the y-axis is the model energy. All the models generated are plotted. Results for the same three targets used in Figure 3 were shown. **(A)**, 1A0R (bound case); **(B)**, 1IZB (bound); **(C)**, 6RLX (bound); **(D)**, 1A0R (unbound case); **(E)**, 1IZB (unbound); **(F)**, 6RLX (unbound).

The energy vs. RMSD plots (Figure 4) also confirms this observation. The energy and RMSD show desired correlation showing funnel-like shape between them for the first two targets, 1A0R (Figure 4A) and 1IZB (Figure 4B). On the other hand, essentially no correlation was observed for 6RLX (Figure 4C); thus, selecting decoys based on the energy did not work in the RL procedure prevented the RL algorithm from discovering lower RMSD models.

In Supplementary Table S6, we showed the number of times lower energy models were discovered during RL-MLZerD runs. For all the targets, about the same number of lower energy models were discovered, which indicates that the reward policy was working similarly also for large complexes with more subunits.

### Unbound docking experiments

Next, we evaluated the performance of the methods on unbound cases (Table 2), which are docking experiments with subunit structures that are not separated from the complex structures. Ideally, experimentally determined subunit structures in an isolated condition would be used but here we used computational models of subunits because genuine unbound structures were not determined and available in PDB. Subunit structure modeling results are shown in Supplementary Table S7. Subunit structures were modelled by the template-based modeling method, MODELLER (Webb and Sali, 2016) if an appropriate template was found for a target by HHpred (Zimmermann et al., 2018). If not, we used structure prediction methods, AttentiveDist (Jain et al., 2021), trRosetta (Du et al., 2021) or I-TASSER (Zheng et al., 2021) The average Cα RMSD of the unbound subunits is 1.46 Å.

**TABLE 2:** Summary of unbound docking results.

PDB-ID	RLMZD	M-LZerD	CombDock
1A0R (3)	3.35 (3.35)	3.24 (2.86)	19.34 (19.34)
6GWJ (3)	11.35 (2.73)	24.23 (4.16)	17.03 (12.94)
1VCB (3)	1.76 (1.58)	12.32 (1.58)	14.58 (14.58)
1A6A (3)	9.39 (5.02)	12.85 (5.37)	15.9 (10.43)
1IOD (3)	6.33 (4.28)	6.14 (4.28)	7.19 (7.19)
1NVV (3)	3.82 (3.82)	23.0 (4.16)	16.24 (12.1)
4YX7 (3)	16.5 (9.68)	16.83 (11.24)	20.02 (16.98)
2GD4 (3)	17.67 (13.07)	18.7 (13.35)	23.69 (13.34)
2H47 (3)	4.54 (2.59)	18.96 (2.54)	21.31 (13.69)
2ASS (3)	4.74 (3.66)	22.7 (8.65)	16.26 (10.79)
1P3Q (3)	7.1 (4.64)	8.68 (6.32)	12.61 (11.11)
1JSU (3)	10.1 (7.26)	15.63 (5.41)	24.82 (18.3)
1EPT (3)	12.68 (8.97)	12.72 (8.84)	18.76 (15.54)
1RHM (4)	3.8 (3.8)	17.56 (3.74)	21.47 (17.17)
1NNU (4)	21.14 (5.83)	19.79 (13.01)	21.37 (17.51)
1QGW (4)	18.74 (11.69)	17.02 (8.48)	20.03 (18.7)
1CYD (4)	3.08 (3.08)	1.50 (1.50)	20.84 (20.84)
1IZB (4)	7.27 (3.64)	10.96 (1.77)	10.12 (9.38)
6MWR (4)	23.05 (12.66)	37.14 (14.59)	22.16 (22.16)
3LL8 (4)	6.90 (6.9)	27.01 (14.28)	31.56 (23.46)
4IHH (4)	25.72 (14.87)	25.33 (18.36)	36.52 (27.1)
6RLX (4)	11.1 (7.15)	11.43 (8.07)	10.92 (10.2)
1GL2 (4)	18.32 (4.94)	31.82 (7.36)	15.78 (15.78)
3UAI (4)	24.09 (9.53)	28.72 (18.72)	24.69 (19.84)
1D1I (5)	8.66 (3.51)	10.62 (1.16)	13.91 (12.59)
1CT1 (5)	11.21 (6.72)	13.15 (9.37)	19.34 (16.2)
1CN3 (5)	1.6 (1.6)	2.31 (2.31)	28.04 (26.19)
1W85 (5)	7.42 (7.42)	7.57 (7.01)	33.64 (28.9)
4FTG (5)	9.27 (4.66)	13.03 (8.06)	13.99 (13.21)
4RT4 (5)	11.82 (10.31)	13.74 (8.35)	16.02 (13.78)
Avg. (Å)	**10.75 (6.30)**	16.16 (7.50)	19.61 (16.31)
≤8.0 Å	**13 (22)**	5 (16)	1 (1)

The best (smallest) RMSD among the top 5 scored models were considered. The best RMSD among all the models generated before clustering is shown in parenthesis at each target. The total number of models generated is provided in Supplementary Table S3. For RL-MLZerD, only the version with a fixed P of 0.6 was shown because it performed better than the Metropolis version in Table 1. The best RMSD within the top 5 ranked models were reported. Notations are the same as Table 1.

On the unbound cases, RL-MLZerD still showed the lowest average RMSD among the three methods with Multi-LZerD and CombDock. Comparing bound and unbound results for each target, we observe that the RMSD increased by more than a couple of angstroms for most of the targets. Particularly, the RMSD went over 10 Å for 16 targets in the unbound docking (Table 2) while there were 3 such cases in bound docking (Table 1). For 10 of the targets, models with an RMSD less than 10 Å were generated, but they were not ranked with in top 5 by the scoring function. The average difference between the bound and unbound docking results was smaller (2.50 Å and 6.30 Å for the bound and unbound cases, respectively) when all the generated models were considered (values are in parentheses in Tables 1, 2) than the best among top 5 scored models were considered. Thus, the difference of subunit structures between the unbound conformation and the bound conformation also affected the scoring.

As before with the bound cases, we analyzed the RL agent behavior in selecting good quality pairwise decoys during the episodes. The unbound counterpart of the first example, 1A0R (Figure 3D), showed similar behavior where good pairwise models were increasingly selected with a usage peaking at 40% around the 3500th episode. It was around this episode that the best RMSD model was first discovered. In the next example 1IZB (Figure 3E), we observed a different pattern from the bound case (Figure 3D). In the bound case we observed an increase of the selection of A-B hits, which was not observed in the unbound case. The usage of hits including for the A-B pair stayed relatively low throughout the episodes. This may be a part of the reason that the best RMSD model found has a larger RMSD than what Multi-LZerD had found. The best RMSD model was generated at around the 3000th episode, when the selection of the A-B hits showed a small peak. Finally, the last example 6RLX (Figure 3F) is also consistent with the bound counterpart (Figure 3C), as we can observe the lower energy model being selected continuously during the episodes as usage grows up to 70%. However, the lower energy pairwise model does not correspond to a good quality model, resulting in 7.15 Å RMSD for the best generated model.

Exploring the relationship between energy and RMSD further reinforces our findings. 1A0R unbound (Figure 4D) is consistent with the bound example as the funnel-like shape is observed. 1IZB unbound (Figure 4E) is also similar to the bound example, with the only difference that no models had an RMSD less than 2 Å. Finally, as we saw for the bound case there is no apparent correlation for 6RLX (Figure 4F).

Similar to the bound experiment, in Supplementary Table S8 we examined how well the RL agent identified the best possible RMSD model from the set of available pairwise unbound decoys. For 60.0% (18/30) of the cases, a model within 1.0 Å RMSD to the best possible RMSD for a complex was generated by RL-MLZerD. The average RMSD difference of between the best possible RMSD and the one achieved was 1.67 Å.

### Interface accuracy

**FIGURE 5:**
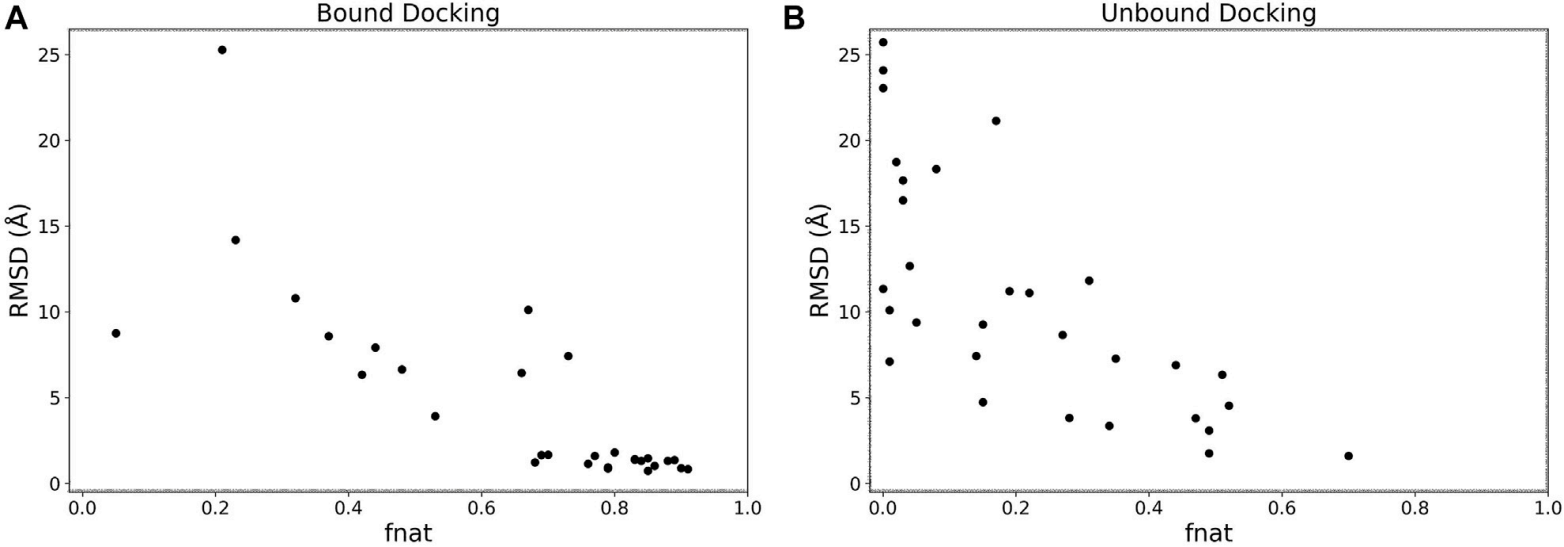
RMSD vs. fnat. The best RMSD (Å) among the top 5 scored models relative to its fnat. **(A)**, the bound docking experiment; **(B)**, the unbound docking experiment.

We analyzed the interface accuracy of generated models in the bound and the unbound docking experiments. We used the fraction of native contacts (fnat) as the metric of the interface accuracy, which is used in CAPRI (Méndez et al., 2003) and defined as the fraction of native residue-residue contacts (within 5 Å from each other) found in the computational model relative to the total number of native contacts. In Figure 5 we plotted the best RMSD among the top 5 scored models and fnat of the same models. Obviously, the two metrics have a correlation as both evaluate the accuracy of models. For the bound cases (Figure 5A), we observe that models with an RMSD less than 4 Å all have high fnat values above 0.7. This is not the case for the unbound cases shown in Figure 5B where fnat values of models with an RMSD less than 4 Å ranged widely from 0.28 to 0.70.

### Comparison with AlphaFold

We further compared the docking performance of RL-MLZerD with AlphaFold (Table 3). Note that rigorous objective comparison is not possible because we do not have information of the training dataset (28 out of the 30 benchmark datasets have a release date that predates the AlphaFold training set cutoff date as shown in Table 1) used to tune parameters of AlphaFold and also because the input information for these programs is different. Therefore, the purpose of this comparison is simply to provide rough idea of their relative performance. We used two versions of AlphaFold, the implementation in the ColabFold website, where input sequences were concatenated with linker sequences (Mirdita et al., 2021) and AlphaFold-Multimer (Evans et al., 2021), which are designed for complex modeling. We ran AlphaFold-Multimer with and without templates. These three AlphaFold methods output five models and the best (smallest) RMSD among them is reported in Table 3 for each target.

**TABLE 3:** Comparison of RL-MLZerD against AlphaFold variations.

PDB-ID	RLMZD (bound)	RLMZD (unbound)	ColabFold	AF-Mult.	AF-Mult. (nt)
1A0R	0.87 (0.87)	3.35 (3.35)	2.21	1.63	1.68
6GWJ	0.88 (0.81)	11.35 (2.73)	0.89	1.8	1.62
1VCB	1.38 (1.33)	1.76 (1.58)	0.86	0.79	0.81
1A6A	0.74 (0.74)	9.39 (5.02)	2.98	1.27	0.59
1IOD	6.33 (1.85)	6.33 (4.28)	4.73	5.81	1.93
1NVV	0.94 (0.94)	3.82 (3.82)	5.08	2.72	3.22
4YX7	1.6 (1.6)	16.5 (9.68)	5.23	1.64	2.55
2GD4	8.75 (1.97)	17.67 (13.07)	3.77	0.85	1.32
2H47	1.14 (1.14)	4.54 (2.59)	7.8	4.47	2.48
2ASS	3.92 (3.92)	4.74 (3.66)	7.27	7.13	7.03
1P3Q	1.37 (1.37)	7.1 (4.64)	13.08	9.44	11.54
1JSU	0.84 (0.84)	10.1 (7.26)	2.41	1.16	1.33
1EPT	1.23 (1.23)	12.68 (8.97)	0.63	0.3	0.76
1RHM	1.67 (1.67)	3.8 (3.8)	0.41	0.31	0.54
1NNU	1.47 (1.47)	21.14 (5.83)	0.80	0.79	1.06
1QGW	14.19 (9.26)	18.74 (11.69)	26.01	14.07	14.1
1CYD	1.66 (1.66)	3.08 (3.08)	0.83	0.60	0.55
1IZB	1.03 (0.95)	7.27 (3.64)	1.69	1.34	10.9
6MWR	25.27 (2.6)	23.05 (12.66)	28.93	20.16	23.35
3LL8	1.31 (1.21)	6.90 (6.9)	16.45	17.8	20.0
4IHH	10.12 (10.12)	25.72 (14.87)	4.31	0.87	0.98
6RLX	8.59 (5.45)	11.1 (7.15)	7.07	0.45	0.43
1GL2	6.64 (2.22)	18.32 (4.94)	0.56	0.37	0.38
3UAI	10.8 (2.97)	24.09 (9.53)	19.94	1.9	1.75
1D1I	7.92 (1.27)	8.66 (3.51)	0.46	0.35	0.34
1CT1	1.32 (1.32)	11.21 (6.72)	7.88	0.60	1.97
1CN3	1.42 (1.15)	1.6 (1.6)	-	0.37	4.50
1W85	7.43 (4.76)	7.42 (7.42)	-	0.50	0.61
4FTG	1.80 (1.80)	9.27 (4.66)	1.69	1.91	1.89
4RT4	6.44 (6.44)	11.82 (10.31)	9.01	2.47	6.16
Avg.(Å)	4.64 (2.50)	10.75 (6.30)	6.54	3.46	4.21
≤8.0 Å	24 (28)	13 (22)	22	26	25

RLMZD (bound), RLMLZD (unbound) reported the lowest RMSD among top 5 scored models in the bound and unbound docking results, respectively (Tables 1, 2). The number in the parentheses is the lowest RMSD observed in the pool of generated models before clustering; AF-Multimer, AlphaFold-Multimer; AF-M (nt), AlphaFold-Multimer without using templates. ColabFold was used on 27th October 2021 at https://colab.research.google.com/github/sokrypton/ColabFold/blob/main/beta/AlphaFold2_advanced.ipynb. AlphaFold-Multimer was downloaded on 4th November 2021 from https://github.com/deepmind/alphafold and ran locally. The smallest RMSD for each target is highlighted in bold. The two cases with hyphen (-) for ColabFold are the instance where the notebook failed to run due to memory limitation (requires >16 GB of GPU memory) on Google Colab.

First, we compare the bound docking result of RL-MLZerD. AlphaFold-Multimer with templates showed the lowest average RMSD of 3.46 Å. When templates were not used in AlphaFold-Multimer, RMSD increased for most of the targets, with some with a large margin, resulting in an average RMSD of 4.21 Å. ColabFold failed to run for 2 targets, and the average of the rest of the targets was 6.54 Å. This average by ColabFold is worse than the bound case results of RL-MLZerD. When RMSD values of individual targets were compared, RL-MLZerD had a lower value than ColabFold for 16 (Krissinel and Henrick, 2007) targets out of 28 targets (in the parenthesis shown is when all the generated models were considered for RL-MLZerD). When compared with AlphaFold-Multimer (with template), RL-MLZerD was better in 11 (Jumper et al., 2021) targets. When no templates were used in AlphaFold-Multimer, RL-MLZerD showed a lower RMSD in 13 (Mirdita et al., 2021) targets. Therefore, although AlphaFold-Multimer can model multimeric complexes with higher accuracy than RL-MLZerD in general, there are a good number of cases where RL-MLZerD can provide better models. Particularly, if we consider the number of targets with an RMSD of less than 8.0 Å, RL-MLZerD had a higher value than AlphaFold-Multimer for 28 targets out of 30 when all generated models were considered. AlphaFold-Multimer had higher value for 26 targets compared to 24 of RL-MLZerD when only top 5 ranking models were considered.

Next, we compare the unbound docking results of RL-MLZerD with AlphaFold-Multimer (no template). The average RMSD of the targets for AlphaFold-Multimer (no template) is lower (4.21 Å) compared to 6.30 Å and 10.75 Å for RL-MLZerD (unbound) when the best generated model or the top 5 models are considered respectively. When individual RMSD values of the targets are considered, RL-MLZerD (unbound) predicted lower RMSDs for 7 targets compared to 23 of AlphaFold-Multimer (no template) when the best predicted model is compared. Finally, the individual cases where either method was able to predict a model less than 8.0 Å is considered, AlphaFold-Multimer can predict less than 8 Å complex structure for 25 targets compared to 22 and 13 for RL-MLZerD (unbound) best model and top 5, respectively.

There are two cases where AlphaFold-Multimer (no-template) failed with a relatively large RMSD, which were worse than unbound docking with RL-MLZerD. 3LL8 (a four-chain complex) is the first case. The structure is a dimer of hetero dimers. AlphaFold-Multimer (no-template) was able to predict the individual dimers correctly. However, the assembly of two dimers went wrong. Two subunits were placed too close to each other causing chain entanglements at the interface. This mis assembly resulted in an RMSD of 20.0 Å. This observation was also true for the regular AlphaFold-Multimer with template. The second case, 1P3Q (a three-chain complex) is a complex of small subunits. The individual structures of two subunits predicted by AlphaFold-Multimer have already some deviations. Partly because of that, only one interface between subunits was correctly built. The resulting RMSD was 11.54 Å.

### Examples of docking models

**FIGURE 6:**
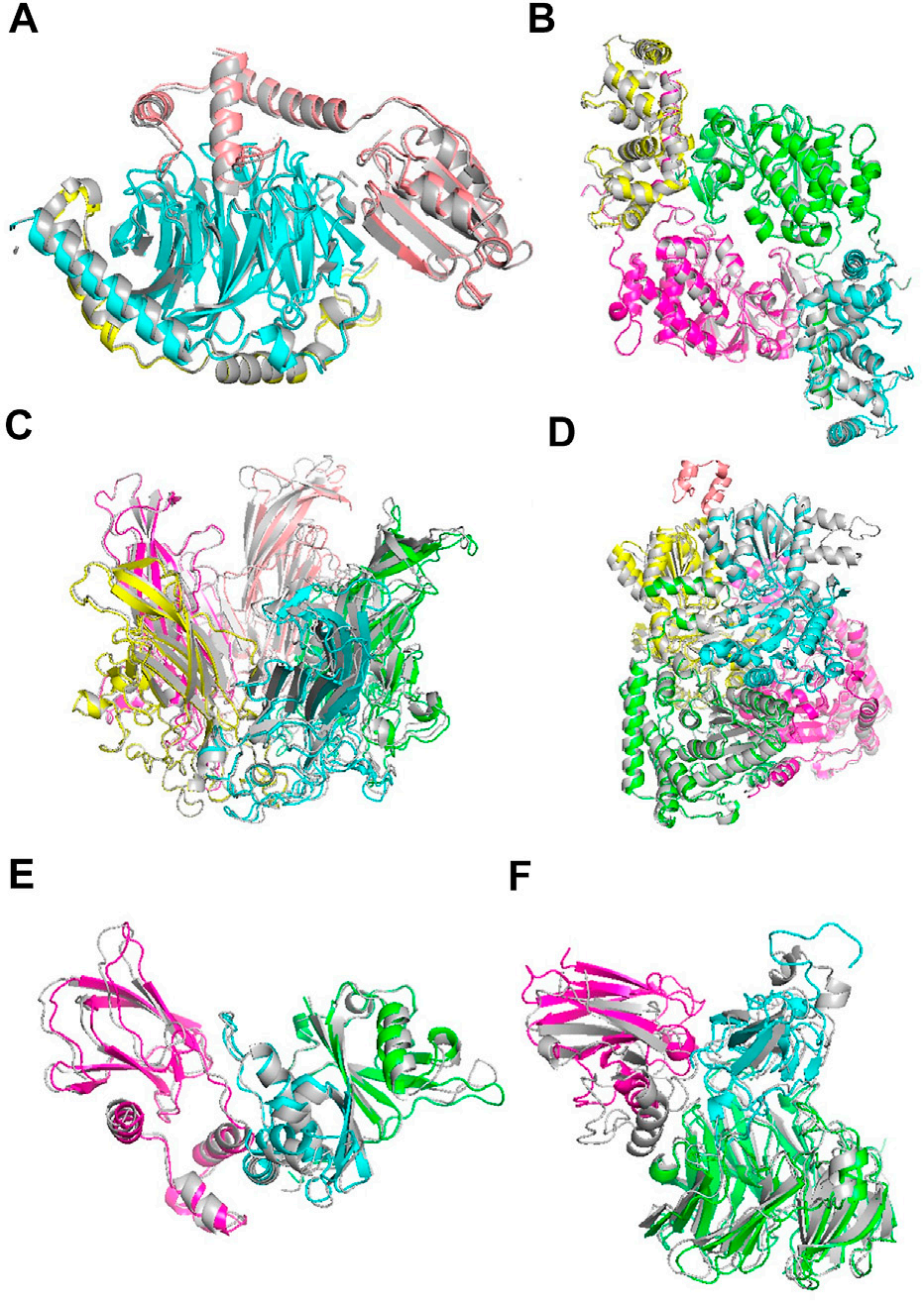
Examples of docking models by RL-MLZerD. The native structure solved by experimental method is in grey, the generated models are shown in colors. Each chain is colored in a different color. The best model among the top 5 models is shown. **(A)**, Transducin Beta-Gamma complex and Phosducin, PDB ID: 1A0R. A 3-chain complex. RMSD, 0.87 Å.**(B)**, Serine/threonine-protein phosphatase 2B catalytic subunit alpha isoform and calcineurin subunit B type 1, PDB ID: 3LL8. A 4 chain complex. RMSD 1.31 Å **(C)**, Capside protein Vp1, PDB ID: 1CN3. A 5-chain complex. RMSD, 1.42 Å. **(D)**, Pyruvate dehydrogenase E1 component subunit alpha and beta and Dihydrolipoyllysine-residue acetyltransferase component of pyruvate dehydrogenase complex, PDB ID: 1W85. A 5-chain complex. RMSD, 7.43 Å **(E)**, Elongin-C and Elongin-B complex with Von Hippel Lindau (VHL), PDB ID: 1VCB. A 3-chain complex. The structures of the individual chains were modelled by MODELLER. RMSD, 1.76 Å **(F)**, Aralkylamine dehydrogenase heavy and light chain and Azurin, PDB ID: 2H47. A 3-chain complex. Subunit structures were modelled by MODELLER. RMSD, 4.54 Å.

Figure 6 shows example cases of the assembled model for each of four targets of increasing chain size. The native structure is in grey and the models are shown with each chain a different color. 1A0R (Figure 6A), 3LL8 (Figure 6B) and 1CN3 (Figure 6C) are assembled almost perfectly with an RMSD of 0.87 Å, 1.31 Å and 1.42 Å, respectively. AF-Multimer struggled with the prediction of 3LL8 having three interaction interfaces and the orientation of chains B and D (magenta and green) predicted incorrectly. 1W85 (Figure 6D) was more challenging due to the small interaction site of chain I (orange; the dihydrolipoyllysine-residue acetyltransferase component of pyruvate dehydrogenase complex). RL-MLZerD agent selected the top of the pocket between chains B (cyan) and D (yellow) as the interaction interface for chain I, instead of the correct interface at the tip of the pocket on chain D. Overall the RMSD of the model is 7.43 Å.

1VCB (Figure 6E) is an example of unbound docking prediction. We used MODELLER to model the three individual chains. The RMSD difference between the model and native structure for the three chain is 1.19 Å, 1.14 Å, and 0.79 Å, respectively (Supplementary Table S7). RL-MLZerD did not have problem in assembling these subunit models resulting an RMSD of 1.76 Å 2H47 (Figure 6F) is another example of the unbound docking prediction. RMSD values between the native structure and the individual chain models by MODELLER are 0.95 Å, 3.73 Å, and 0.54 Å for chain A, B, C, respectively. Despite the high RMSD difference between the native and modelled chain B (3.73 Å), RL-MLZerD was still able to model the complex structure with an RMSD of 4.54 Å.

### Docking order prediction

In this section, we explore the RL-MLZerD model post-docking phase. The objective is to explore the state tree value to infer the assembled order of the subunits. We hypothesized that the path with the highest $v_{i}$ value in the assembly state reflects the biological pathway of how the individual chains combine to form a complex.

This study is important because understanding the complex formation process is critical for several applications. One such application is targeted drug development where we can develop a drug that interrupts the complex formation after a certain subunit is formed. This targeted disruption is only possible if we know the pathway at which the complex is formed. There are experimental methods available for determining such pathways, but these methods are usually expensive. Having a computation approach will be very useful for experimentalists and it will help foster such drug development methodologies. The assembly order of a protein complex can be predicted successfully for most of the cases by considering the buried surface area (BSA) of subunits when the tertiary structure of the complex is known as shown in earlier works by Teichmann et al. (Levy et al., 2008; Marsh et al., 2013). The prediction simply orders the assembly of the complex based on the subunits with the largest BSA. One can think of the BSA approach as an estimation of the binding free energy where large BSA corresponds to lower binding free energy and is hence more favorable to assembly first in the process of complex formation. However, the key limitation to this approach is that it requires the known complex structure and cannot be applied when the subunits are solved separately. We show in Table 4 that RL-MLZerD can perform the prediction without knowing the native structure, by only using the accumulated knowledge from generated episodes.

**TABLE 4:** Docking order prediction results.

PDB-ID	Pathway	RLMZD (bound)	RLMZD (unbound)	Largest BSA	RLMZD (bound) step	RLMZD (unbound) step	BSA step
1A0R	BG > BGP	BG > BGP	BG > BGP	BG > BGP	1/1	1/1	1/1
6GWJ	BD > BDK	BD > BDK	BK > BKD	BK > BKD	1/1	0/1	0/1
1VCB	AB > ABC	AB > ABC	AB > ABC	AB > ABC	1/1	1/1	1/1
1A6A	AB > ABC	AB > ABC	AB > ABC	AB > ABC	1/1	1/1	1/1
1IOD	AB > ABG	AG > AGB	AB > ABG	AB > ABG	0/1	1/1	1/1
4YX7	AB > ABC	AB > ABC	AB > ABC	AB > ABC	1/1	1/1	1/1
2H47	AB > ABC	AB > ABC	AB > ABC	AB > ABC	1/1	1/1	1/1
2GD4	HL > HIL	HI > HIL	HI > HIL	HI > HIL	0/1	0/1	0/1
2ASS	AB > ABC	AB > ABC	AB > ABC	AB > ABC	1/1	1/1	1/1
1P3Q	QR > QRV	QR > QRV	QR > QRV	QR > QRV	1/1	1/1	1/1
1JSU	AB > ABC	AB > ABC	AB > ABC	AC > ACB	1/1	1/1	0/1
1QGW	AC > ACBD	BD > BDA > BDAC	BD > BDC > BDCA	AC > BD > ACBD	2*/2	2*/2	2/2
6MWR	AB > ABCD	AB > ABC > ABCD	AB > ABD > ABDC	CD > AB > ABCD	2*/2	2*/2	2/2
1W85	AA’BB’ > AA’BB’I	BD > BDA > BDAC > BDACI	AB > ABC > ABCD > ABCDI	BD > AC > BDAC > BDACI	1/1	1/1	1/1
4RT4	ABCD > ABCDE	BE > BED > BEDA > BEDAC	BE > BED > BEDA > BEDAC	AB > CD > ABCD > ABCDE	0/1	0/1	1/1
Total correct predictions					14/17	14/17	14/17

PDB-ID is the ID of the protein complex. Pathway is the correct order of the protein complex assemble. RLMZD (bound), RL-MLZD (unbound) is the RL-MLZerD assembly order prediction for the protein complex. Largest BSA is the assembly order predicted by considering the size of the interface of each subunit in the native protein complex. RLMZD (bound, unbound) step and Largest BSA step shows the number of steps predicted correctly by each method. We put asterisk (*) for the prediction results of 1QGW and 6MWR, whose known assembly paths include a parallel assembly step of two subcomplexes. For these two complexes, RL-MLZerD predicted a sequential assembly with one submit added at a time to the complex due to its algorithm design (a tree-based approach), which could be considered as correct because the predictions include all the correct assembly steps.

Table 4 shows the result of docking order prediction. Predictions were made for 15 targets which have evidence of the docking order in the literature. There are 4 categories of such evidence; experimental evidence, biological inference, model of assembly, and authors’ discussion. This evidence follows the approach from previous work from our lab Path-LZerD (Peterson et al., 2018b). Evidence of these 15 targets is provided in Supplementary Information S1. Evidence was classified as biological inference when the order of the assembly can be reasonably inferred from the function of each subunit. Experimental evidence indicates when actual experiment, such as co-immunoprecipitation of subcomplexes, were performed. Model of assembly indicates that the assembly pathway has been proposed in a publication. Finally, author’s discussion indicates when the assemble order is discussed in a publication.

For the 15 targets, which have 17 assembly steps in total, considering the BSAs of subunits predicted 14 steps correctly. RL-MLZerD (bound or unbound) was able to predict all the steps for the assembly order of 12 targets out of 15. RL-MLZerD predicted the same number of steps as the BSA-based approach, 14 out of 17 steps (82.4%), without knowing the tertiary structure of the complexes. The docking order prediction is an interesting by-product of the RL-MLZerD agent exploration as it was able to determine the assemble pathway without explicitly incorporating the information into the model. On a similar dataset of 13 targets with 3–5 chains, Path-LZerD among all its modes achieved at best 69.2% prediction accuracy.

## Discussion

In this work we have proposed and developed a multimeric protein docking method using reinforcement learning. We demonstrated the ability of the RL agent to exploit and explore different states and actions to assemble models with an average RMSD of 2.50 Å for bound and 6.30 Å for unbound cases across 30 targets. RL-MLZerD showed a higher accuracy than the existing methods we compared against, except for AlphaFold-Multimer on unbound cases. We found that RL is better suited for exploring the docking space than the genetic algorithm used in Multi-LZerD because RL is able to identify individual correct decoys through multiple episode runs, which can effectively shrink the search space as the number of runs is increased.

To the best of our knowledge, this is the first time the RL was adapted for the exploration of protein docking conformation search. RL resembles multiple attempts of assembly paths. Through running multiple episodes, RL-MLZerD was able to accumulate knowledge of preferred pairwise decoys and assembly orders that were more likely to lead to near native complex structures. By considering the assembly states of the highest accumulated score, we were able to predict the assembly orders of protein complexes, which is a notable novel finding of this work. Such direct prediction of the assembly path simultaneous with complex structure prediction is not possible by other existing multiple protein docking methods.

From the viewpoint of the RL architecture, RL-MLZerD uses a unique architecture where the goal of an episode is a moving target, a better energy complex structure than so far explored. This is very different from a regular RL task where reward is given when an agent reaches a fix goal. This new RL design is an important contribution of this work.

Looking into a future direction, this work has shown that RL can be useful in identifying preferred protein interactions to build up a larger system in a biologically meaningful order. This approach could be further extended to model a larger system including larger protein complexes with more subunits. It could be also applied to elucidate biomolecular interactions in a cell that include small chemical ligands, proteins, nucleic acids, and membrane. To scale up the system to handle, we could consider higher order interactions, i.e. interactions with more than two proteins (biomolecules) and adopt a more advanced algorithm such as a combinations of deep learning and RL (deep RL).

## Data Availability

Publicly available datasets were analyzed in this study. This data can be found here: Source code for RL-MLZerD is available at https://github.com/kiharalab/RL-MLZerD. The dataset used in this work is also made available at https://doi.org/10.5281/zenodo.6629912.

## References

[B1] Aytuna A. S. Gursoy A. Keskin O. (2005). Prediction of protein-protein interactions by combining structure and sequence conservation in protein interfaces. Bioinformatics 21, 2850–2855. 10.1093/bioinformatics/bti443 15855251

[B2] Berman H. M. (2000). The protein data bank. Nucleic Acids Res. 28, 235–242. 10.1093/nar/28.1.235 10592235 PMC102472

[B3] Christoffer C. Terashi G. Shin W. Aderinwale T. Peterson L. Verburgt J. (2020). Performance and enhancement of the LZerD protein assembly pipeline in CAPRI 38‐46. Proteins. 88, 948–961. 10.1002/prot.25850 31697428 PMC7685511

[B4] Dominguez C. Boelens R. Amjj Bonvin. Haddock (2003). Haddock: A protein-protein docking approach based on biochemical or biophysical information. J. Am. Chem. Soc. 125, 1731–1737. 10.1021/ja026939x 12580598

[B5] Du Z. Su H. Wang W. Ye L. Wei H. Peng Z. (2021). The trRosetta server for fast and accurate protein structure prediction. Nat. Protoc. 16, 5634–5651. 10.1038/s41596-021-00628-9 34759384

[B6] Esquivel-Rodríguez J. Yang Y. D. Kihara D. (2012). Multi-LZerD: Multiple protein docking for asymmetric complexes. Proteins 80, 1818–1833. 10.1002/prot.24079 22488467 PMC3370124

[B7] Evans R. O’Neill M. Pritzel A. Antropova N. Senior A. W. Green T. (2021). Protein complex prediction with AlphaFold-Multimer. bioRxiv.

[B8] Huang S-Y. Zou X. (2011). Statistical mechanics-based method to extract atomic distance-dependent potentials from protein structures. Proteins 79, 2648–2661. 10.1002/prot.23086 21732421 PMC11108592

[B9] Inbar Y. Benyamini H. Nussinov R. Wolfson H. J. (2005). Prediction of multimolecular assemblies by multiple docking. J. Mol. Biol. 349, 435–447. 10.1016/j.jmb.2005.03.039 15890207

[B10] Jain A. Terashi G. Kagaya Y. Maddhuri Venkata Subramaniya S. R. Christoffer C. Kihara D. (2021). Analyzing effect of quadruple multiple sequence alignments on deep learning based protein inter-residue distance prediction. Sci. Rep. 11, 7574–7613. 10.1038/s41598-021-87204-z 33828153 PMC8027171

[B11] Jumper J. Evans R. Pritzel A. Green T. Figurnov M. Ronneberger O. (2021). Highly accurate protein structure prediction with AlphaFold. Nature 596, 583–589. 10.1038/s41586-021-03819-2 34265844 PMC8371605

[B12] Kocsis L. Szepesvári C. (2006). Bandit based monte-carlo planning. Lect. Notes Comput. Sci. 2006, 282–293. 10.1007/11871842_29

[B13] Kozakov D. Hall D. R. Xia B. Porter K. A. Padhorny D. Yueh C. (2017). The ClusPro web server for protein–protein docking. Nat. Protoc. 12, 255–278. 10.1038/nprot.2016.169 28079879 PMC5540229

[B14] Krissinel E. Henrick K. (2007). Inference of macromolecular assemblies from crystalline state. J. Mol. Biol. 372, 774–797. 10.1016/j.jmb.2007.05.022 17681537

[B15] Kundrotas P. J. Vakser I. A. (2010). Accuracy of protein-protein binding sites in high-throughput template-based modeling. PLoS Comput. Biol. 6, e1000727. 10.1371/journal.pcbi.1000727 20369011 PMC2848539

[B16] Lensink M. F. Nadzirin N. Velankar S. Wodak S. J. (2020). Modeling protein‐protein, protein‐peptide, and protein‐oligosaccharide complexes: CAPRI 7th edition. Proteins. 88, 916–938. 10.1002/prot.25870 31886916

[B17] Levy E. D. Erba E. B. Robinson C. v. Teichmann S. A. (2008). Assembly reflects evolution of protein complexes. Nature 453, 1262–1265. 10.1038/nature06942 18563089 PMC2658002

[B18] Marsh J. A. Hernández H. Hall Z. Ahnert S. E. Perica T. Robinson C. V. (2013). Protein complexes are under evolutionary selection to assemble via ordered pathways. Cell 153, 461–470. 10.1016/j.cell.2013.02.044 23582331 PMC4009401

[B19] Méndez R. Leplae R. de Maria L. Wodak S. J. (2003). Assessment of blind predictions of protein-protein interactions: Current status of docking methods. Proteins 52, 51–67. 10.1002/prot.10393 12784368

[B20] Mirdita M. Ovchinnikov S. Steinegger M. (2021). ColabFold-Making protein folding accessible to all. BioRxiv. 10.1038/s41592-022-01488-1 PMC9184281 35637307

[B21] Moal I. H. Bates P. A. (2010). SwarmDock and the use of normal modes in protein-protein docking. Int. J. Mol. Sci. 11, 3623–3648. 10.3390/ijms11103623 21152290 PMC2996808

[B22] Olechnovič K. Venclovas Č. (2017). VoroMQA: Assessment of protein structure quality using interatomic contact areas. Proteins 85, 1131–1145. 10.1002/prot.25278 28263393

[B23] Pereira J. Simpkin A. J. Hartmann M. D. Rigden D. J. Keegan R. M. An Lupas (2021). High‐accuracy protein structure prediction in CASP14. Proteins 89, 1687–1699. 10.1002/prot.26171 34218458

[B24] Peterson L. X. Shin W. Kim H. Kihara D. (2018). Improved performance in CAPRI round 37 using LZerD docking and template‐based modeling with combined scoring functions. Proteins. 86, 311–320. 10.1002/prot.25376 28845596 PMC5820220

[B25] Peterson L. X. Togawa Y. Esquivel-Rodriguez J. Terashi G. Christoffer C. Roy A. (2018). Modeling the assembly order of multimeric heteroprotein complexes. PLoS Comput. Biol. 14, e1005937. 10.1371/journal.pcbi.1005937 29329283 PMC5785014

[B26] Pierce B. Tong W. Weng. M-Zdock Z. (2005). M-ZDOCK: A grid-based approach for cn symmetric multimer docking. Bioinformatics 21, 1472–1478. 10.1093/bioinformatics/bti229 15613396

[B27] Popov P. Ritchie D. W. DockTrina S. Grudinin. (2014). DockTrina: Docking triangular protein trimers. Proteins 82, 34–44. 10.1002/prot.24344 23775700

[B28] Ritchie D. W. Grudinin S. (2016). Spherical polar Fourier assembly of protein complexes with arbitrary point group symmetry. J. Appl. Crystallogr. 49, 158–167. 10.1107/s1600576715022931

[B29] Schneidman-Duhovny D. Inbar Y. Nussinov R. Wolfson H. J. (2005). Geometry-based flexible and symmetric protein docking. Proteins 60, 224–231. 10.1002/prot.20562 15981269

[B30] Sutton R. S. Barto A. G. (1998). Introduction to reinforcement learning. Cambridge, Massachusetts, US: MIT press Cambridge.

[B31] Terashi G. Shibuya T. Takeda-Shitaka M. (2012). LB3D: A protein three-dimensional substructure search program based on the lower bound of a root mean square deviation value. J. Comput. Biol. 19, 493–503. 10.1089/cmb.2011.0230 22509779

[B32] Tokic M. (2010). “Adaptive -greedy exploration in reinforcement learning based on value differences,” in Annual Conference on Artificial Intelligence, Atlanta, Georgia, USA, July 11–15, 2010.

[B33] Venkatraman V. Yang Y. D. Sael L. Kihara D. (2009). Protein-protein docking using region-based 3D Zernike descriptors. BMC Bioinforma. 10, 407–421. 10.1186/1471-2105-10-407 PMC2800122 20003235

[B34] Webb B. Sali A. (2016). Comparative protein structure modeling using MODELLER. Curr. Protoc. Bioinforma. 54, 5–6. 10.1002/cpbi.3 PMC5031415 27322406

[B35] Zheng W. Zhang C. Li Y. Pearce R. Bell E. W. Zhang Y. (2021). Folding non-homologous proteins by coupling deep-learning contact maps with I-TASSER assembly simulations. Cell Rep. Methods 1, 100014. 10.1016/j.crmeth.2021.100014 34355210 PMC8336924

[B36] Zhou H. Goap J. Skolnick. (2011). Goap: A generalized orientation-dependent, all-atom statistical potential for protein structure prediction. Biophys. J. 101, 2043–2052. 10.1016/j.bpj.2011.09.012 22004759 PMC3192975

[B37] Zhou H. Zhou Y. (2009). Distance-scaled, finite ideal-gas reference state improves structure-derived potentials of mean force for structure selection and stability prediction. Protein Sci. 11, 2714–2726. 10.1110/ps.0217002 PMC2373736 12381853

[B38] Zimmermann L. Stephens A. Nam S-Z. Rau D. Kübler J. Lozajic M. (2018). A completely reimplemented MPI bioinformatics toolkit with a new HHpred server at its core. J. Mol. Biol. 430, 2237–2243. 10.1016/j.jmb.2017.12.007 29258817

