# Supplementary material for: RL-MLZerD: Multimeric protein docking using reinforcement learning
Source: Front Mol Biosci. 2022 Aug 26;9:969394. doi: 10.3389/fmolb.2022.969394 (PMC9459051; doi:10.3389/fmolb.2022.969394)
Supplement: Supplementary file 1 [file DataSheet1.pdf]

Supplementary Material

Supplementary Table S1. Benchmark dataset details information

PDB-ID	Name	# of chains	Residue counts	Resolution (Å)	Release Date
1A0R	Transducin Beta-Gamma complex and Phosducin	3	650	2.80	1998-12-30
6GWJ	EKC/KEOPS subunit GON7, LAGE3 with Human OSGEP	3	584	1.95	2019-07-03
1VCB	Elongin-C and Elongin-B complex with Von Hippel Lindau(VHL)	3	1560	2.70	1999-04-21
1A6A	HLA class II histocompatibility antigen Alpha, Beta and Gamma.	3	378	2.75	1998-05-27
1IOD	Snaclec anticoagulant subunit A and B with Coagulation Factor X	3	296	2.30	2001-06-27
1NVV	GTPase HRas and Son of sevenless homolog 1	3	813	2.18	2003-04-01
4YX7	Surface presentation of antigens protein SpaO with Endolysin	3	680	2.00	2015-06-03
2H47	Aralkylamine dehydrogenase heavy and light chain and Azurin	3	2228	2.60	2006-11-21
2GD4	Coagulation factor X and Antithrombin-III	3	1484	3.30	2006-05-09
2ASS	S-phase kinase-associated protein 1 and 2 with Cyclin-dependent kinases regulatory subunit 1	3	564	3.00	2005-10-18
1P3Q	Vacuolar protein sorting-associated protein 9 and Polyubiquitin-C	3	260	1.70	2003-06-24
1JSU	Cyclin-A2, Cyclin-dependent kinase 2 and Cyclin-dependent kinase inhibitor 1B	3	642	2.30	1997-07-29
1EPT	Trypsin	3	223	1.80	1995-02-07
1RHM	The Complex Of Caspase-3	4	498	2.50	2004-05-11
1NNU	Enoyl-Acyl-Carrier-Protein Reductase.	4	578	2.50	2003-02-25
1QGW	Phycoerythrin alpha 2 and 3 chain, chloroplastic with B-phycoerythrin beta chain	4	497	1.63	1999-05-19
1CYD	Carbonyl Reductase [Nadph] 2	4	976	1.80	1996-10-14
1IZB	Insulin complex	4	102	2.00	1993-10-31
6MWR	G7 Gamma/Delta chain of T cell receptor, Major histocompatibility complex class I-related gene protein and Beta-2-microglobulin	4	850	3.30	2019-12-18

3LL8	Serine/threonine-protein phosphatase 2B catalytic subunit alpha isoform and calcineurin subunit B type 1	4	1035	2.00	2011-01-12
4IHH	UDP-3-O-(3-hydroxymyristoyl) glucosamine N-acyltransferase and Acyl Carrier Protein	4	2568	2.13	2013-11-13
6RLX	Prorelaxin H2	4	104	1.50	1993-10-31
1GL2	Syntaxin 7 and 8,Vesicle-associated membrane protein 8, and Vesicle transport through interaction with t-SNAREs homolog 1B	4	260	1.90	2002-01-15
3UAI	Protein SHQ1,H/ACA ribonucleoprotein complex subunit GAR1, NOP10, and CBF5	4	938	3.06	2011-12-14
1D1I	Shiga-Like Toxin 1 Subunit B	5	345	1.70	2000-09-20
1CT1	Cholera enterotoxin subunit B	5	515	2.30	1997-10-15
1CN3	Capside protein Vp1	5	1444	2.20	1999-06-09
1W85	Pyruvate dehydrogenase E1 component subunit alpha and beta and Dihydrolipoyllysine-residue acetyltransferase component of pyruvate dehydrogenase complex	5	2866	2.00	2004-11-02
4FTG	Protein S100-A10, Annexin A2 and Neuroblast differentiation-associated protein AHNAK	5	248	2.51	2013-01-02
4RT4	Protein dpy-30 homolog and COMPASS component Bre2	5	294	2.00	2015-10-07

Supplementary Table S2. Results with different hyper-parameter combinations.

PDB-ID	# of Chains	Norm. Factor	Full Reward	Rejected Model	Penalty	Best RMSD (Å)	Worst RMSD (Å)	Average RMSD (Å)
1A0R	3	Any	Any	Any	Any	0.87	0.87	0.87
1IOD	3	Any	50	5	-10	2.51	6.18	5.1
1A6A	3	Any	Any	Any	Any	0.74	0.77	0.74
6RLX	4	6	100	5	-2	3.76	6.6	5.54
1RHM	4	2	10	10	-2	1.43	4.79	1.96
1CYD	4	8	50	10	-8	0.84	1.54	1.04
1W85	5	6	100	1	-2	3.86	5.4	4.55
4FTG	5	6	10	1	-2	1.24	1.94	1.36

1CT1	5	4	100	10	-2	1.23	1.76	1.46
Majority		6	100	10	-2			

Combinations of four parameters used in RL were tested on three targets each for 3-chain, 4-chain, and 5-chain complexes. The four parameters explored were the normalization factor (Eq. 2), the full reward, the reward given to a model that did not pass the Metropolis criterion, and the penalty for a model with too many clashes. We tested 2, 4, 6, and 8 for the normalization factor; 100, 50, and 10 for the full reward; 10, 5, and 1 for the reward to a rejected model; -2, -8 and -10 for the penalty. For each parameter combination, we recorded the best (smallest) RMSD among all the generated model. Then, in the table we reported the best and worst RMSD among all the parameter combinations as well as the average RMSD value. The majority reports the value that were selected for the largest number of times among the nine targets.

Supplementary Table S3. Total number of models generated by RL-MLZerD.

PDB-ID	Bound	Unbound
1A0R	5731	5959
6GWJ	5931	10165
1VCB	7781	7351
1A6A	7835	8783
1IOD	4514	4892
1NVV	8513	8516
4YX7	7692	9034
2H47	7347	9639
2GD4	11756	11120
2ASS	9831	10666
1P3Q	10189	10995
1JSU	9386	10379
1EPT	7886	14845
1RHM	3609	2352
1NNU	9213	7338
1QGW	5879	2952
1CYD	3376	1102
1IZB	7860	5033
6MWR	10324	11247
3LL8	4563	3659
4IHH	2394	6637
6RLX	6895	4945
1GL2	5533	5660
3UAI	16661	18578
1D1I	4583	8064
1CT1	8617	14168
1CN3	3190	2961
1W85	5015	4116
4FTG	10167	17441
4RT4	1768	1286

Here we report the total number of models generated by RL-MLZerD for each target.

Supplementary Figure S1. The best RMSD within top 5 scored models relative to the secondary structure contents.

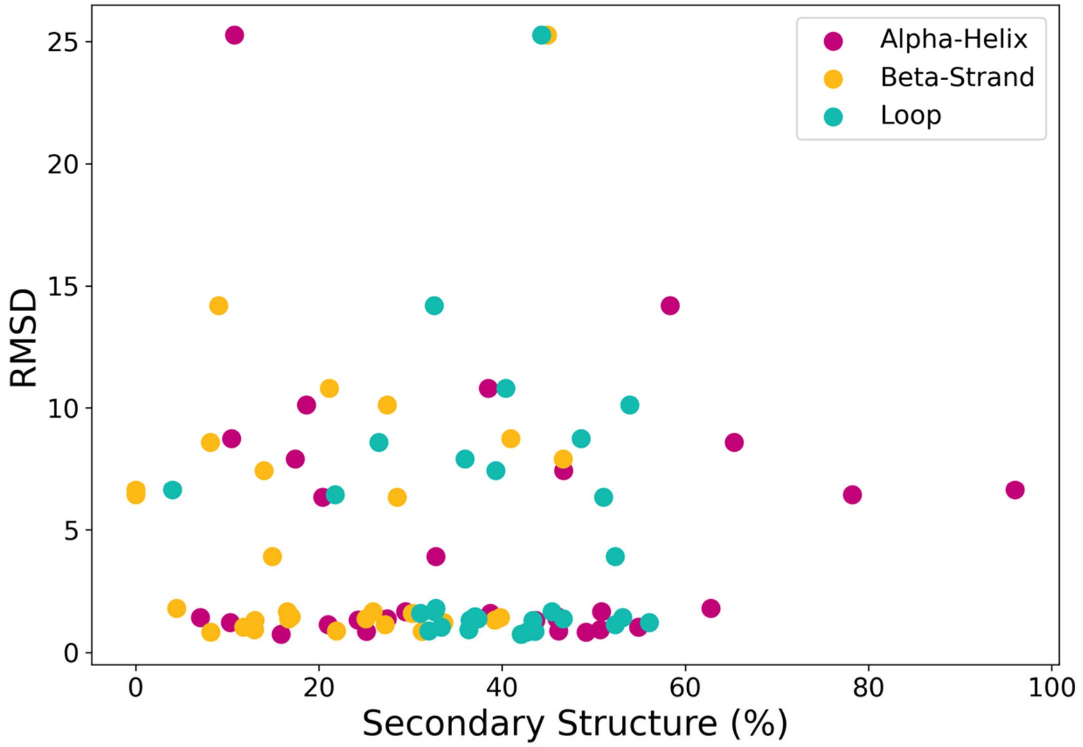

The best RMSD (Å) among the top 5 scored models were plotted relative to the percentage of residues in α helices, β strands, and loops. Thus, there are three data points for each target complex. The secondary structure was defined with DSSP (1). Residues classified as H, G, and I were considered as α helices, those classified as E and B were considered as β strands, and the rest were grouped as loop. There is no clear correlation between RMSD and the secondary structure of the structure.

Supplementary Table S4. Evaluating the effect of ions in modeling accuracy.

PDB-ID	Ion Location	Best RMSD (Å)	Top 5 best RMSD (Å)
1IOD	Interface	1.85	6.33
1NVV	Interface	0.94	0.94
2ASS	Interface	3.92	3.92
1JSU	Interface	0.84	0.84
1QGW	Interface	9.26	14.19
1W85	Interface	4.76	7.43
6GWJ	Non Interface	0.81	0.88
2H47	Non Interface	1.97	8.75
2GD4	Non Interface	1.14	1.14
1EPT	Non Interface	1.23	1.23
1IZB	Non Interface	0.95	1.03
3LL8	Non Interface	1.21	1.31
1CT1	Non Interface	1.31	1.32
1A0R	Not Present	0.87	0.87
1VCB	Not Present	1.33	1.38
1A6A	Not Present	0.74	0.74
4YX7	Not Present	1.60	1.60
1P3Q	Not Present	1.37	1.37
1RHM	Not Present	1.67	1.67
1NNU	Not Present	1.47	1.47
1CYD	Not Present	1.66	1.66
6MWR	Not Present	2.60	25.27
4IHH	Not Present	10.1	10.12
6RLX	Not Present	5.45	8.59
1GL2	Not Present	2.22	6.64
3UAI	Not Present	2.97	10.8
1D1I	Not Present	1.27	7.92
1CN3	Not Present	1.15	1.42
4FTG	Not Present	1.80	1.80
4RT4	Not Present	6.44	6.44

We investigated the effect of ions in modeling the complex structure. There are 13 protein complexes in our dataset that contain at least one ion. The complex structures were grouped into three; the group one includes structures with an ion at the interface (within 5 Å to heavy atoms from two or more chains in the complex); the second group are cases where an ion exist in the structure but not at the interface; and the third group are for structures with no ion.
We evaluated statistical significance of difference of the RMSD value distribution. First, we considered group one and two, i.e. complexes that have ions at the interface and complexes with ions but not at the interface. We performed the Wilcoxon-Mann-Whitney (2) test using the overall best RMSD and the top 5 best RMSD. We used Wilcoxon-Mann-Whitney because the variance between the two groups was significantly different according to the Bartlett test (3) The p-value of the test was 0.14 and 0.26, respectively, for the overall best RMSD and the top 5 best RMSD. Therefore, the difference is not significant at p-value of 0.05.

Next, we compare group one and the rest of the dataset (groups two and three), i.e. complexes with an ion at the interface and the rest. We used t-test (4) this time because the variance assumption is met (by Bartlett test (3) the p-value was 0.24 and 0.76 for the overall best RMSD and the top 5 best RMSD, respectively). The p-value of the t-test was 0.22 and 0.63 for the overall best RMSD and the top 5 best RMSD, respectively. Based on these results, we concluded that ion presence does not significantly affect the modeling accuracy of RL-MLZerD.

Supplementary Table S5. Approximate of the best possible RMSD from the pairwise decoy pools used in bound experiment.

PDB-ID	Best possible RMSD (Å)	RL-MLZerD (Å)	Best RMSD Identified	Within 1.0 Å
1A0R	0.87	0.87	Yes	Yes
6GWJ	0.81	0.81	Yes	Yes
1VCB	0.72	1.33	No	Yes
1A6A	0.74	0.74	Yes	Yes
1IOD	2.49	1.85	Yes	Yes
1NVV	0.8	0.94	No	Yes
4YX7	1.54	1.6	No	Yes
2H47	1.23	1.97	No	Yes
2GD4	1.14	1.14	Yes	Yes
2ASS	3.92	3.92	Yes	Yes
1P3Q	1.37	1.37	Yes	Yes
1JSU	0.8	0.84	No	Yes
1EPT	1.23	1.23	Yes	Yes
1RHM	1.37	1.67	No	Yes
1NNU	1.17	1.47	No	Yes
1QGW	2.68	9.26	No	No
1CYD	0.87	1.66	No	Yes
1IZB	0.86	0.95	No	Yes
6MWR	1.84	2.6	No	Yes
3LL8	1.1	1.21	No	Yes
4IHH	3.9	10.12	No	No
6RLX	4.46	5.45	No	Yes
1GL2	1.18	2.22	No	No
3UAI	0.97	2.97	No	No
1D1I	1.27	1.27	Yes	Yes
1CT1	1.32	1.32	Yes	Yes
1CN3	1.15	1.15	Yes	Yes
1W85	2.3	4.76	No	No
4FTG	1.1	1.80	No	Yes
4RT4	6.1	6.44	No	Yes
Avg. (Å)	1.71	2.50		

The best possible RMSD that can be generated from pairwise decoy set used was approximated as follows: We first selected the top 5 best RMSD models in the pairwise decoy pool of 1,000

decoys for each subunit pair and then generated all possible combinations of the top 5 best RMSD decoys and chose the complex with the best RMSD among them. The RMSD is reported in the column named the Best possible RMSD. When compared with the best RMSD actually generated by RL-MLZerD, the approximated best RMSD (or better) was identified among all the generated models for 36.7% (11/30) cases. For 83.3% (25/30) cases, a model within 1.0 Å RMSD was generated by RL-MLZerD with an average RMSD difference of 0.79 Å.

Supplementary Table S6. The number of models with the new lower energy found in RL runs.

PDB-ID	Unique Energies Discovered
1A0R	9
6GWJ	10
1VCB	14
1A6A	13
1IOD	10
1NVV	6
4YX7	10
2H47	6
2GD4	9
2ASS	8
1P3Q	17
1JSU	5
1EPT	8
1RHM	7
1NNU	10
1QGW	12
1CYD	11
1IZB	11
6MWR	8
3LL8	11
4IHH	10
6RLX	11
1GL2	21
3UAI	11
1D1I	11
1CT1	13
1CN3	13
1W85	7
4FTG	10
4RT4	12

Episodes of RL-MLZerD with the fixed probability were analyzed.

Supplementary Table S7. Subunit structure modeling for unbound docking.

PDB-ID	Chain	Unbound	Seq. Identity (%)	RMSD (Å)	Modelling Method
1A0R	B	5KDO_B	98.2	1.29	MODELLER
	G	5KDO_G	86.2	3.20	MODELLER
	P	2TRC_P	79.2	1.39	MODELLER
6GWJ	B	4WXA_B	26.7	2.42	MODELLER
	D	4WXA_E	17.5	2.29	MODELLER
	K	2IVN_A	47.3	1.82	MODELLER
1VCB	A	4AJY_B	89.0	1.19	MODELLER
	B	1LQB_B	78.6	1.14	MODELLER
	C	4AJY_V	92.5	0.79	MODELLER
1A6A	A	6BLQ_A	55.8	1.06	MODELLER
	B	1FNE_D	77.5	1.97	MODELLER
	C	1MUJ_C	100	2.17	MODELLER
1IOD	A	1J34_A	73.6	0.90	MODELLER
	B	1J34_B	87.0	0.57	MODELLER
	G	1J34_C	36.2	2.50	MODELLER
1NVV	Q	2CE2_X	98.2	1.71	MODELLER
	R	6MQT_D	99.4	1.03	MODELLER
	S	6D56_B	99.8	1.01	MODELLER
4YX7	A	-	-	2.22	trRosetta
	B	-	-	1.75	trRosetta
	C	-	-	4.95	I-Tasser
2GD4	H	2JKH_A	99.1	0.76	MODELLER
	I	3KCG_I	99.3	1.79	MODELLER
	L	-	-	2.00	trRosetta
2H47	A	2OIZ_A	98.9	0.95	MODELLER
	B	2OIZ_D	88.6	3.73	MODELLER
	C	2IAA_C	100	0.54	MODELLER
2ASS	A	6M90_B	93.0	2.19	MODELLER
	B	7B5M_T	98.8	1.42	MODELLER
	C	3QY2_A	50.0	1.78	MODELLER
1P3Q	Q	-	-	3.96	trRosetta
	R	-	-	3.77	I-Tasser
	V	-	-	0.82	AttentiveDist
1JSU	A	-	-	2.59	I-Tasser
	B	4EOJ_D	99.2	0.54	MODELLER
	C	-	-	2.24	trRosetta
1EPT	A	2R0L_A	44.2	1.67	MODELLER
	B	4IGD_A	29.3	2.64	MODELLER
	C	5R43_C	47.1	2.03	MODELLER
1RHM	A	2DKO_A	100	1.46	MODELLER
	B	2DKO_B	100	0.50	MODELLER
	C	2DKO_C	100	1.46	MODELLER
	D	2DKO_D	100	0.51	MODELLER

1NNU	A	3LT0_A	98.7	0.68	MODELLER
	B	3LT0_A	98.7	0.68	MODELLER
	C	1NHW_C	96.7	0.55	MODELLER
	D	1NHW_C	96.7	0.52	MODELLER
1QGW	A	1XG0_A	98.7	2.60	MODELLER
	B	1XG0_B	100	1.81	MODELLER
	C	1XG0_C	97.7	0.20	MODELLER
	D	1XG0_C	97.7	1.80	MODELLER
1CYD	A	3D3W_A	67.6	0.76	MODELLER
	B	3D3W_A	67.6	0.77	MODELLER
	C	3D3W_A	67.6	0.77	MODELLER
	D	3D3W_A	67.6	0.77	MODELLER
1IZB	A	3W7Y_A	100	0.64	MODELLER
	B	3W7Y_B	96.7	0.88	MODELLER
	C	3W7Y_C	100	0.54	MODELLER
	D	3W7Y_D	96.7	1.93	MODELLER
6MWR	A	6PUD_C	99.2	1.40	MODELLER
	B	3OV6_A	23.2	2.67	MODELLER
	C	1HXM_B	91.6	1.43	MODELLER
	D	4LFH_D	91.8	1.37	MODELLER
3LL8	A	4F0Z_A	100	0.80	MODELLER
	B	4F0Z_B	94.2	1.74	MODELLER
	C	4F0Z_A	100	0.84	MODELLER
	D	4F0Z_B	94.2	1.78	MODELLER
4IHH	A	6P89_A	99.1	2.28	MODELLER
	B	6P89_A	100	0.91	MODELLER
	C	6P89_A	99.4	2.21	MODELLER
	G	-	-	1.02	AttentiveDist
6RLX	A	1ZEI_C	20.8	1.78	MODELLER
	B	2MV1_B	100	1.89	MODELLER
	C	1ZEI_C	20.8	1.76	MODELLER
	D	2MV1_B	100	1.34	MODELLER
1GL2	A	-	-	1.22	AttentiveDist
	B	-	-	1.72	AttentiveDist
	C	-	-	0.91	AttentiveDist
	D	-	-	0.87	AttentiveDist
3UAI	A	-	-	4.02	I-Tasser
	B	-	-	1.55	trRosetta
	C	3U28_C	100	0.57	MODELLER
	D	-	-	1.19	trRosetta
1D1I	A	1C4Q_A	98.6	0.17	MODELLER
	B	1C4Q_A	98.6	0.18	MODELLER
	C	1C4Q_A	98.6	0.17	MODELLER
	D	1C4Q_A	98.6	0.17	MODELLER
	E	1C4Q_A	98.6	0.17	MODELLER
1CT1	D	5ELB_D	99.0	0.73	MODELLER
	E	5ELB_D	99.0	0.76	MODELLER
	F	5ELB_D	99.0	0.44	MODELLER
	G	5ELB_D	99.0	0.47	MODELLER

	H	5ELB_D	99.0	0.77	MODELLER
1CN3	A	5CPU_C	97.5	1.03	MODELLER
	B	5CPU_C	97.5	1.03	MODELLER
	C	5CPU_C	97.5	1.03	MODELLER
	D	5CPU_C	97.5	1.03	MODELLER
	E	5CPU_C	97.5	1.03	MODELLER
1W85	A	1W88_A	94.4	2.52	MODELLER
	B	1W88_B	100	0.24	MODELLER
	C	1W88_C	93.4	2.82	MODELLER
	D	1W88_D	100	0.20	MODELLER
	1	1W88_I	100	0.38	MODELLER
4FTG	A	1A4P_A	96.7	0.93	MODELLER
	B	1A4P_A	96.7	1.03	MODELLER
	C	6DGP_C	23.1	2.48	MODELLER
	D	6DGP_C	23.1	2.94	MODELLER
	E	1L4D_B	25.0	6.55	MODELLER
4RT4	A	4RTA_A	96.2	0.66	MODELLER
	B	4RTA_A	96.2	0.66	MODELLER
	C	4RTA_A	96.2	1.10	MODELLER
	D	4RTA_A	96.2	1.26	MODELLER
	E	6VEN_O	85.7	1.78	MODELLER

If appropriate template structures for template-based modeling was found by HHpred, we performed modeling with MODELLER. The sequence identity and the PDB ID was shown in that case. If not, we used structure prediction methods, AttentiveDist, trRosetta or I-Tasser for modeling. C-alpha RMSD of modelled subunits are reported.

Supplementary Table S8. Approximate of the best possible RMSD from the pairwise decoy pools used in unbound experiment.

PDB-ID	Best possible RMSD (Å)	RL-MLZerD (Å)	Best RMSD Identified	Within 1.0 Å
1A0R	2.86	3.35	No	Yes
6GWJ	2.26	2.73	No	Yes
1VCB	1.51	1.58	No	Yes
1A6A	4.31	5.02	No	Yes
1IOD	4.5	4.28	Yes	Yes
1NVV	2.97	3.82	No	Yes
4YX7	7.44	9.68	No	No
2H47	12.66	13.07	No	Yes
2GD4	2.46	2.59	No	Yes
2ASS	3.66	3.66	Yes	Yes
1P3Q	3.88	4.64	No	Yes
1JSU	2.92	7.26	No	No
1EPT	8.77	8.97	No	Yes
1RHM	3.54	3.8	No	Yes
1NNU	1.8	5.83	No	No
1QGW	4.46	11.69	No	No
1CYD	2.79	3.08	No	Yes
1IZB	1.56	3.64	No	No
6MWR	11.14	12.66	No	No
3LL8	6.52	6.9	No	Yes
4IHH	7.45	14.87	No	No
6RLX	3.1	7.15	No	No
1GL2	2.52	4.94	No	No
3UAI	7.71	9.53	No	No
1D1I	3.51	3.51	Yes	Yes
1CT1	5.81	6.72	No	Yes
1CN3	1.6	1.6	Yes	Yes
1W85	6.53	7.42	No	Yes
4FTG	3.05	4.66	No	No
4RT4	5.69	10.31	No	No
Avg. (Å)	4.63	6.30		

The RMSD of the best possible RMSD that can be generated from pairwise decoy set is reported in the column named the Best possible RMSD. When compared with the best RMSD actually generated by RL-MLZerD in the unbound experiment, the approximated best RMSD (or better) was identified among all the generated models for 13.33% (4/30) cases. For 60.0% (18/30) cases, a model within 1.0 Å RMSD was generated by RL-MLZerD with an average RMSD difference of 1.67 Å.

Supplementary Information 1. Evidence of assemble order of the targets.

Only the proteins with assembly order information are discussed.

1A0R (3 chains)
Heterotrimeric complex of phosducin/transducin βγ (5). Phosducin (chain P) binds to the transducin β γ dimer (chains B and G) in a regulatory fashion. The association between the transducin β and γ subunits is very strong (5, 6). Thus, the assembly order is BG>BGP.
Evidence type: Biological inference is used to determine the interaction between transducin βγ (subunit B and G), Experimental results from (5, 6) supports the interaction of the transducin βγ and elucidate the impact of phosducin on the subcomplex. Hence the evidence of this assemble order is Biological inference (B) and Experimental evidence (E).

6GWJ (3 chains)
Fungal Gon7 (subunit D) was shown to be an intrinsic disordered protein that adopts a well-defined structure upon complex formation with (LAGE3) (subunit B) (7), therefore, subunit B and D form a complex first. Then, subunit K is assembled next as due to the linear arrangement of the five subunits (GON7, which is subunit D; LAGE3, which is subunit B; OSGEP, subunit K;) of the eukaryotic KEOPS complex in which the three subunits are a part. (8–10). Thus, the assembly order is BD> BDK.
Evidence type: Experimental evidence from (7) highlights the complex formation between subunit B and D. Biological inference is then used to determine subunit K interaction with the rest of the subcomplex because of the linear nature of the interaction as shown in (8–10). Hence the evidence for this assemble is Biological inference (B) and Experimental evidence (E).

1VCB (3 chains)
The VHL-elonginC-elonginB structure (11). This complex is between elongin B and C (chains A and B) and von Hippel-Lindau (VHL) tumor suppressor (chain C). Elongin B and C separately have poor interaction with VHL (12). Thus, the assembly order is AB>ABC.
Evidence type: Biological inference is used to determine subunit A and C subcomplex interaction because both are part of a larger complex. Experimental evidence from (12) discuss the implication of the VHL (subunit C) on subunit A and B. The authors further proposed a model of the complex where VHL is interacting with elongin-BC in absent of elongin-A. Finally due to the modeling order of the whole complex, we determine that the assembly evidence of the complex is Biological inference (B), Experimental evidence (E) and Model of assembly (M).

1A6A (3 chains)
The HLA class II histocompatibility membrane glycoprotein (alpha and beta, which are subunit A and B) forms a complex (13) and HLA class II histocompatibility gamma chain plays a critical role in MHC class II antigen (subunit C) processing by stabilizing peptide-free class II alpha/beta heterodimers (14). Thus, the assembly order is AB> ABC
Evidence type: Biological Inference (B)

1IOD (3 chains)

The crystal structure of X-bp (Snaclec anticoagulant protein subunit A and B) in complex with the Gla domain peptide of factor X (subunit C) at a 2.3Å resolution. The two patches of the Gla domain essential for membrane binding are buried in the complex (subunit A and B) formation (15). Thus, the assembly order is AB> ABG.
Evidence type: Biological Inference (B) because subunit A and B are from the same organism and (15) showed Factor X binding (subunit C) to form a complex thereafter.

4YX7 (3 chains)
Numerous studies have shown the SpaO homologues FliM (subunit A) and FliN (subunit B) to form a robust, stable ring (the 'C-ring') at the cytoplasmic face of the basal body (16). Furthermore, Notti R, et al. (17) showed a heterotypic interaction between SPOA domains (subunit A and B) serving as a scaffold for sorting platform assembly in both injectisome (subunit C) and flagellar T3SS. Solution nuclear magnetic resonance (NMR) data supports the crystallographic model, and structure-guided mutagenesis shows that this interaction is necessary for formation of the SpaO–OrgB–InvC complex. Thus, the assembly order is AB > ABC.
Evidence type: Biological inference is used to determine the complex between subunit A and B and results from (16) supports this conclusion. Experimental evidence from (17) show the interaction modality between subunit C and the rest of the complex. Hence the assemble evidence for the complex is Biological Inference (B) and Experimental evidence (E).

2H47 (3 chains)
The subunit of aromatic amine dehydrogenase (AADH) light and heavy chain (subunit A and B) forms a complex (18). The transient kinetic studies of the electron transfer reaction from AADH to azurin (subunit C) was performed (19). Thus, the assembly order is AB>ABC.
Evidence type: Experimental evidence (E) due to the experimental results of (18) and (19).

2GD4 (3 chains)
Coagulation Factor X is a glycoprotein composed of a heavy chain (subunit H) and light chain (subunit L) held together by a single disulfide bond (20). Antithrombin AT (subunit I) interacts with factors IXa (subunit H) which form a complex between pentasaccharide-activated AT and fXa. (21). Thus, the assembly order is HL> HIL
Evidence type: Experimental evidence (E) due to the experimental results of (20) and (21).

2ASS (3 chains)
Skp1-Skp2 (subunit A and B) form a complex (22). Cks1 (subunit C) binds to the catalytic subunit (Skp2) of the cyclin dependent kinases and is essential for their biological function (23). Thus, the assemble order is AB> ABC.
Evidence type: Biological inference is used to determine subunit A and B complex interaction, experimental results from (22) supports this conclusion. Experimental results from (23) was used to determine the interaction of subunit C with the other sub-complex. Therefore, the evidence for this target is Biological inference (B) and Experimental evidence (E).

1P3Q (3 chains)

The Vps9p CUE domain (subunit Q and R) forms extensive dimer contacts across a crystallographic two-fold axis (24). The CUE domain of the sorting protein Vps9p binds directly to monoubiquitin (subunit V) (25). Thus, the assemble order is QR>QRV.
Evidence type: Biological inference is used to determine subunit Q and R complex interaction, experimental results from (24) supports this conclusion. Experimental results from (25) was used to determine the interaction of subunit V with the other sub-complex. Therefore, the evidence for this target is Biological inference (B) and Experimental evidence (E).

1JSU (3 chains)
cyclin A (subunit B) and Cdk2 (subunit A) form a complex, Cyclin-dependent kinase inhibitor 1b (subunit C) interacts with both cyclin A (subunit B) and Cdk2 (subunit A) (26, 27). Thus, the assemble order is AB>ABC.
Evidence type: Experimental evidence (E) from both manuscripts suggest the initial complex formation of subunit A and B and the inhibitor activity of subunit C.

1QGW (4 chains)
The overall structure of Phycoerythrin 545 $\alpha_1\alpha_2\beta\beta$ dimer forms a boat-shaped molecule. The dimer is formed by the two tightly linked $\alpha_1\beta$ monomers (subunit A and C) and $\alpha_2\beta$ monomers (subunit B and D). The α subunits are not identical with the major sequence and structural difference residing in the C-terminal regions. The C-terminal extension of α_1 is important in the chromophore binding. Almost all the protein-protein contact between the two monomers is mediated through the α subunits. A C_α tracing of the dimer forms the asymmetric unit of the complex (28). Thus, the assembly order is AC> ACBD.
Evidence type: The author discussion from (28) elucidate the interaction between the subunits, hence the assembly evidence is Author Discussion (AD).

6MWR (4 chains)
Beta-2-microglobulim (subunit B) is a component of the class I major histocompatibility complex (MHC) (subunit A), hence both form a complex. γδ T cell receptors form a complex (subunit C and D) (29, 30). The two subcomplexes come together to form a single complex as it is show that the diversity of the T cell is restricted by MHC class I related molecule (30). Thus, the assemble order is AB> ABCD or CD>ABCD.
Evidence type: Biological inference is used to determine the interaction between subunit A and B. Experimental results from (29, 30) show the interaction between subunit C and D. Finally, experimental results from (30) highlight the impact of both individual subunits coming together to form a single complex. Hence the evidence for this assemble is Biological inference (B) and Experimental evidence (E).

1W85 (5 chains)
There is a crystal structure where pyruvate dehydrogenase E1(D180N, E183Q) bound to the peripheral sub- unit binding domain of E2 (31) . This structure consists of two copies of the pyruvate dehydrogenase E1 α subunit (chains A and C in the PDB file, denoted as A and A', respectively), two copies of the β subunit (chains B and D, denoted as B and B'), and the peripheral subunit binding domain of E2 (chain I). Because the structure of E1 can be solved in the absence

of E2 (32), it is expected that E1 will assemble before E2 binds. Thus, the assembly order is $AA'BB'>AA'BB'I$.
Evidence type: Biological inference (B) as supported by (31) and (32).

4RT4 (5 chains)
The subunits (A, B, C and D) of Protein dpy-30 are an important part of the MLL1/MLL core complex (33). The dpy complex directly interact with the Compass component BRE2 (subunit E) through its Dpy30-binding motif (34). Thus, the assembly order is ABCD>ABCDE.
Evidence type: Biological inference is used to determine the interaction between subunit A, B, C and D by drawing inference from (33). Experimental evidence from (34) is then used to determine subunit E interaction with the rest of the complex. Hence the evidence for this assemble is Biological inference (B) and Experimental evidence (E).

References

1. W Kabsch; C Sander. Dictionary of protein secondary structure: Pattern recognition of hydrogen-bonded and geometrical features. *Biopolymers* 22, 2577–2637 (1983)
2. HB Mann; DR Whitney. On a Test of Whether one of Two Random Variables is Stochastically Larger than the Other. *The Annals of Mathematical Statistics* 18, 50–60 (1947)
3. Maurice Stevenson Bartlett. Properties of sufficiency and statistical tests. *Proceedings of the Royal Society of London Series A - Mathematical and Physical Sciences* 160, 268–282 (1937)
4. Student. THE PROBABLE ERROR OF A MEAN. *Biometrika* 6, 1–25 (1908)
5. A Loew; Y-K Ho; T Blundell; B Bax. Phosducin induces a structural change in transducin βγ. *Structure* 6, 1007–1019 (1998)
6. J Dingus; JD Hildebrandt. Synthesis and Assembly of G Protein βγ Dimers: Comparison of In Vitro and In Vivo Studies. In: (2012)
7. W Zhang; B Collinet; M Graille; M-C Daugeron; N Lazar; D Libri; D Durand; H van Tilbeurgh. Crystal structures of the Gon7/Pcc1 and Bud32/Cgi121 complexes provide a model for the complete yeast KEOPS complex. *Nucleic Acids Research* 43, 3358–3372 (2015)
8. A Costessi; N Mahrour; V Sharma; R Stunnenberg; MA Stoel; E Tijchon; JW Conaway; RC Conaway; HG Stunnenberg. The Human EKC/KEOPS Complex Is Recruited to Cullin2 Ubiquitin Ligases by the Human Tumour Antigen PRAME. *PLoS ONE* 7, e42822 (2012)
9. DYL Mao; D Neculai; M Downey; S Orlicky; YZ Haffani; DF Ceccarelli; JSL Ho; RK Szilard; W Zhang; CS Ho; L Wan; C Fares; S Rumpel; I Kurinov; CH Arrowsmith; D Durocher; F Sicheri. Atomic Structure of the KEOPS Complex: An Ancient Protein Kinase-Containing Molecular Machine. *Molecular Cell* 32, 259–275 (2008)
10. LCK Wan; P Maisonneuve; RK Szilard; J-P Lambert; TF Ng; N Manczyk; H Huang; R Laister; AA Caudy; A-C Gingras; D Durocher; F Sicheri. Proteomic analysis of the human KEOPS complex identifies C14ORF142 as a core subunit homologous to yeast Gon7. *Nucleic Acids Research* 45, 805–817 (2017)
11. CE Stebbins; WG Kaelin; NP Pavletich. Structure of the VHL-ElonginC-ElonginB Complex: Implications for VHL Tumor Suppressor Function. *Science (1979)* 284, 455–461 (1999)

12. DR Duan; A Pause; WH Burgess; T Aso; DYT Chen; KP Garrett; RC Conaway; JW Conaway; WM Linehan; RD Klausner. Inhibition of Transcription Elongation by the VHL Tumor Suppressor Protein. *Science (1979)* 269, 1402–1406 (1995)
13. P Ghosh; M Amaya; E Mellins; DC Wiley. The structure of an intermediate in class II MHC maturation: CLIP bound to HLA-DR3. *Nature* 378, 457–462 (1995)
14. JM Riberdy; JR Newcomb; MJ Surman; JA Barbosa; P Cresswell. HLA-DR molecules from an antigen-processing mutant cell line are associated with invariant chain peptides. *Nature* 360, 474–7 (1992)
15. H Mizuno; Z Fujimoto; H Atoda; T Morita. Crystal structure of an anticoagulant protein in complex with the Gla domain of factor X. *Proceedings of the National Academy of Sciences* 98, 7230–7234 (2001)
16. R Zhao; N Pathak; H Jaffe; TS Reese; S Khan. FliN is a Major Structural Protein of the C-ring in theSalmonella typhimuriumFlagellar Basal Body. *Journal of Molecular Biology* 261, 195–208 (1996)
17. RQ Notti; S Bhattacharya; M Lilic; CE Stebbins. A common assembly module in injectisome and flagellar type III secretion sorting platforms. *Nature Communications* 6, 7125 (2015)
18. N Sukumar; Z Chen; D Ferrari; A Merli; GL Rossi; HD Bellamy; A Chistoserdov; VL Davidson; FS Mathews. Crystal Structure of an Electron Transfer Complex between Aromatic Amine Dehydrogenase and Azurin from *Alcaligenes faecalis* ,. *Biochemistry* 45, 13500–13510 (2006)
19. P ROSEN; M SEGAL; I PECHT. Electron Transfer between Azurin from Alcaligenes faecalis and Cytochrome c551 from Pseudomonas aeruginosa. *European Journal of Biochemistry* 120, 339–344 (1981)
20. BA McMullen; K Fujikawa; W Kisiel; T Sasagawa; WN Howald; EY Kwa; B Weinstein. Complete amino acid sequence of the light chain of human blood coagulation factor X: evidence for identification of residue 63 as .beta.-hydroxyaspartic acid. *Biochemistry* 22, 2875–2884 (1983)
21. DJD Johnson; W Li; TE Adams; JA Huntington. Antithrombin–S195A factor Xa-heparin structure reveals the allosteric mechanism of antithrombin activation. *The EMBO Journal* 25, 2029–2037 (2006)
22. BA Schulman; AC Carrano; PD Jeffrey; Z Bowen; ERE Kinnucan; MS Finnin; SJ Elledge; JW Harper; M Pagano; NP Pavletich. Insights into SCF ubiquitin ligases from the structure of the Skp1–Skp2 complex. *Nature* 408, 381–386 (2000)
23. B Hao; N Zheng; BA Schulman; G Wu; JJ Miller; M Pagano; NP Pavletich. Structural Basis of the Cks1-Dependent Recognition of p27Kip1 by the SCFSkp2 Ubiquitin Ligase. *Molecular Cell* 20, 9–19 (2005)
24. G Prag; S Misra; EA Jones; R Ghirlando; BA Davies; BF Horazdovsky; JH Hurley. Mechanism of Ubiquitin Recognition by the CUE Domain of Vps9p. *Cell* 113, 609–620 (2003)
25. SC Shih. A ubiquitin-binding motif required for intramolecular monoubiquitylation, the CUE domain. *The EMBO Journal* 22, 1273–1281 (2003)
26. M Pagano; R Pepperkok; F Verde; W Ansorge; G Draetta. Cyclin A is required at two points in the human cell cycle. *The EMBO Journal* 11, 961–971 (1992)

27. DA McGrath; B Fifield; AH Marceau; S Tripathi; LA Porter; SM Rubin. Structural basis of divergent cyclin-dependent kinase activation by Spy1/ <scp>RINGO</scp> proteins. *The EMBO Journal* 36, 2251–2262 (2017)
28. KE Wilk; SJ Harrop; L Jankova; D Edler; G Keenan; F Sharples; RG Hiller; PMG Curmi. Evolution of a light-harvesting protein by addition of new subunits and rearrangement of conserved elements: Crystal structure of a cryptophyte phycoerythrin at 1.63-Å resolution. *Proceedings of the National Academy of Sciences* 96, 8901–8906 (1999)
29. AJ Corbett; SBG Eckle; RW Birkinshaw; L Liu; O Patel; J Mahony; Z Chen; R Reantragoon; B Meehan; H Cao; NA Williamson; RA Strugnell; D van Sinderen; JYW Mak; DP Fairlie; L Kjer-Nielsen; J Rossjohn; J McCluskey. T-cell activation by transitory neo-antigens derived from distinct microbial pathways. *Nature* 509, 361–365 (2014)
30. NA Gherardin; AN Keller; RE Woolley; J Le Nours; DS Ritchie; PJ Neeson; RW Birkinshaw; SBG Eckle; JN Waddington; L Liu; DP Fairlie; AP Uldrich; DG Pellicci; J McCluskey; DI Godfrey; J Rossjohn. Diversity of T Cells Restricted by the MHC Class I-Related Molecule MR1 Facilitates Differential Antigen Recognition. *Immunity* 44, 32–45 (2016)
31. RAW Frank; CM Titman; JV Pratap; BF Luisi; RN Perham. A Molecular Switch and Proton Wire Synchronize the Active Sites in Thiamine Enzymes. *Science (1979)* 306, 872–876 (2004)
32. M Kato; RM Wynn; JL Chuang; S-C Tso; M Machius; J Li; DT Chuang. Structural Basis for Inactivation of the Human Pyruvate Dehydrogenase Complex by Phosphorylation: Role of Disordered Phosphorylation Loops. *Structure* 16, 1849–1859 (2008)
33. A Patel; V Dharmarajan; VE Vought; MS Cosgrove. On the Mechanism of Multiple Lysine Methylation by the Human Mixed Lineage Leukemia Protein-1 (MLL1) Core Complex. *Journal of Biological Chemistry* 284, 24242–24256 (2009)
34. H Zhang; M Li; Y Gao; C Jia; X Pan; P Cao; X Zhao; J Zhang; W Chang. Structural implications of Dpy30 oligomerization for MLL/SET1 COMPASS H3K4 trimethylation. *Protein & Cell* 6, 147–151 (2015)
